# Effects of Web-Based Single-Session Growth Mindset Interventions for Reducing Adolescent Anxiety: Four-Armed Randomized Controlled Trial

**DOI:** 10.2196/63500

**Published:** 2025-04-18

**Authors:** Shimin Zhu, Yuxi Hu, Di Qi, Samson Tse, Ko Ling Chan, Jessica Sun, Paul Lee

**Affiliations:** 1 Department of Applied Social Sciences The Hong Kong Polytechnic University Hong Kong China (Hong Kong); 2 Department of Social Work and Social Administration The University of Hong Kong Hong Kong China (Hong Kong); 3 Kowloon Hospital Child & Adolescent Mental Health Centre Hong Kong China (Hong Kong); 4 Southampton Clinical Trials Unit University of Southampton Southampton United Kingdom

**Keywords:** belief-in-change, growth mindset, mental health, secondary school students, brief intervention, randomized controlled trial

## Abstract

**Background:**

Anxiety disorders are the most common mental health conditions worldwide, yet 65% of those affected do not access services. The high prevalence of anxiety and the low rate of intervention uptake highlight the urgent need to develop timely, scalable, and effective interventions suitable for adolescents. This study adapted existing single-session interventions (SSIs) to further develop an SSI focused on a growth mindset regarding negative emotions for adolescent mental health.

**Objective:**

The study aims to compare the effectiveness of 4 SSIs, SSI of a growth mindset for anxiety (SIGMA), SIGMA with boosters (SIGMA-Booster), SSI of a growth mindset of personality (SSIGP), and an active control group (support therapy [ST]), in reducing adolescent anxiety.

**Methods:**

Classes from each secondary school were randomized to 1 of 4 intervention conditions: SIGMA, SIGMA-Booster, SSIGP, or ST. Each intervention took approximately 45 minutes online. Participants reported on anxiety symptoms (primary outcome), depressive symptoms, suicidal/self-harming thoughts, perceived control, hopelessness, attitude toward help-seeking, and psychological well-being (secondary outcomes) at preintervention, 2-week follow-up, and 8-week follow-up. Participants also completed a feedback scale postintervention. Generalized estimating equations were used to examine the effectiveness of the SSIs.

**Results:**

A total of 731 adolescents from 7 secondary schools were randomized. The intent-to-treat analysis found a significant decrease in anxiety symptoms. The mean and 95% CI at baseline were 6.8 (6.0-7.6) for SIGMA-Booster, 6.5 (5.8-7.3) for SIGMA, 7.0 (6.2-7.7) for SSIGP, and 6.9 (6.1-7.7) for ST. At the 2-week follow-up, the mean and 95% CI were 5.9 (5.1-6.7) for SIGMA-Booster, 5.7 (4.9-6.5) for SIGMA, 5.4 (4.6-6.2) for SSIGP, and 5.7 (4.9-6.4) for ST. At the 8-week follow-up, the mean and 95% CI were 5.9 (5.1-6.7) for SIGMA-Booster, 5.3 (4.5-6.0) for SIGMA, 5.6 (4.8-6.4) for SSIGP, and 5.8 (5.1-6.6) for ST. These reductions were observed across all 4 groups. Moderation analysis found that participants with higher motivation for change, higher baseline anxiety scores, and fixed mindsets showed greater improvements in anxiety symptoms. Most participants (459/731, 62.8%) viewed the feasibility and acceptability of the SSIs positively.

**Conclusions:**

The SSI for all 4 groups was effective in reducing anxiety and depression among adolescents over 8 weeks. Our data suggest the potential benefits of brief web-based interventions for adolescents, which could serve as scalable, destigmatized, and cost-effective alternatives to school-based programs. The intervention effects may have been underestimated, as this study did not exclude adolescents with minimal or no anxiety symptoms. Future studies should focus on the specific effects of interventions for adolescents with varying levels of anxiety symptoms.

**Trial Registration:**

ClinicalTrials.gov NCT05027880; https://clinicaltrials.gov/ct2/show/NCT05027880

## Introduction

### Background

Anxiety is one of the leading causes of illness and disability among adolescents aged 10-19 years [1]. Approximately 6.5% of adolescents worldwide and 6.9% in Hong Kong have been diagnosed with anxiety disorders [2,3]. In Hong Kong, for example, 1 in 4 secondary school students experienced high subclinical anxiety symptoms over a 3-month period, requiring clinical intervention [4]. Based on prevalence rates and local youth population data [5], it is estimated that approximately 85,000 secondary school students in Hong Kong require help and intervention for anxiety symptoms.

However, an estimated 65% of individuals with generalized anxiety disorder in Hong Kong did not access mental health services [6]. Among those who sought help, the median waiting time from symptom onset to receiving public child and adolescent psychiatric services was 58 weeks. Meanwhile, the cost of private treatment was reported to be HK $3000 (US $386) per monthly consultation, making it unaffordable for many families, who were left with no choice but to wait for public services [7]. Existing approaches, such as clinic-based treatments provided by highly trained mental health professionals, face significant limitations, including lengthy waitlists, high costs, and challenges in large-scale dissemination. This traditional setting further restricts access to services in special circumstances, such as during the COVID-19 pandemic [8]. Moreover, adolescents with mental health symptoms are particularly vulnerable to stigma and discrimination and may be reluctant to seek school-based interventions [9]. Even among youth who access care, most drop out prematurely, completing only 3-4 therapy sessions on average [10]. While natural remission without treatment may occur, it is uncommon [11]. Given these challenges, there is a clear need to develop briefer, scalable, nonstigmatizing, and youth-friendly interventions for adolescents with general anxiety symptoms. A brief intervention that fosters insight or reduces anxiety about mental health symptoms may help alleviate mental health problems and support remission.

Single-session interventions (SSIs) are structured programs designed to involve only 1 visit or encounter with a clinic, provider, or program [12]. These interventions can function as stand-alone treatments or as adjuncts to clinical services. Notably, research has shown that the number of sessions is not related to the magnitude of the treatment effect [13]. As a very brief intervention consisting of just 1 session, SSIs have demonstrated a relatively substantial effect on youth psychiatric problems. A previous meta-analysis [14] reported a mean postintervention effect size of 0.32 (Hedges *g*), with the largest effect size observed for anxiety (0.56). Although the effects of SSIs were moderated by follow-up length, with smaller effect sizes observed in follow-ups exceeding 13 weeks [14], Schleider and Weisz [15] still found significant improvements in youth depression and perceived behavioral control following growth mindset SSIs, with effects lasting up to 9 months compared with an active control. They also found that enhancing belief in personality change improved treatment access for adolescent depression [16], although no significant changes were recorded for general anxiety, social anxiety, or conduct problems [17]. Walton and Wilson [18,19] emphasized the importance of precise interventions that target the underlying psychological processes contributing to social or psychological problems, as well as the need for an adaptive context to maximize the potency of brief interventions. Thus, the effectiveness of SSIs is closely tied to their content (what to intervene) and implementation strategies (how to intervene).

Given the substantial evidence linking fixed mindsets to youth mental health problems [20] and the positive effects of growth mindset SSIs on anxiety-related outcomes [21], this study developed and examined the efficacy of growth mindset SSIs for adolescent anxiety in the Chinese context. Although some cultural adaptations of growth mindset SSIs have been made for non-Western populations, such as Indian adolescents [22], few studies have examined growth mindset SSIs in the Chinese context, particularly for anxiety-related outcomes [23]. We aimed to advance the existing literature by implementing and comparing different domains of growth mindset and developing implementation strategies for SSIs among Chinese adolescents. First, this study developed the SSI of a growth mindset for anxiety (SIGMA). As fixed mindsets about negative emotions (beliefs that one’s negative emotions cannot change) have been closely associated with adolescent depression and anxiety [24,25], interventions that promote growth mindsets about negative emotions (beliefs in the changeability of one’s negative emotions) may help alleviate worry and anxiety in adolescents. Second, to examine the effectiveness of SIGMA, we adapted the existing SSIs into Chinese and compared them with SSI of a growth mindset of personality (SSIGP) and support therapy (ST) [15,26]. Third, we collected feedback from social workers and counselors based on the principle of patient and public involvement, and they suggested that boosters could help strengthen the effectiveness of brief interventions. Thus, we designed booster reminders for SIGMA and examined whether reinforcing the core messages of the intervention with boosters would strengthen its effectiveness. A deviation from the 3-arm intervention protocol is that we increased the sample size of participants receiving SIGMA with booster (SIGMA-Booster) messages, establishing it as an independent group. As a result, the SIGMA-Booster group and the SIGMA group now have sample sizes equivalent to the other 2 groups (SSIGP and ST), rather than selecting only half of the original SIGMA group to receive booster messages. We proposed this 4-arm randomized controlled trial to provide evidence on the effectiveness of SIGMA (including SIGMA and SIGMA-Booster) and compare it against the existing growth mindset intervention (SSIGP) and support theory as an active control. Beyond its research significance, the study’s findings could have broad implications for mental health care practice and policy. If the SSIs in this study are found to be effective, they could be scaled up in schools and community settings, offering a cost-effective and accessible solution to address youth mental health needs. This could reduce the burden on overstretched traditional mental health services and inform public health policies, such as integrating SSIs into school mental health programs or national strategies.

### Objectives

The primary objective of the study was to evaluate the effectiveness of a single-session growth mindset intervention for negative emotions (abbreviated as SIGMA) in reducing general anxiety symptoms among secondary school students.

The secondary objective was to compare the effectiveness of the aforementioned programs on secondary outcomes, including reductions in depressive symptoms, suicidal/self-harming thoughts, and hopelessness, as well as increases in perceived control over emotions, attitudes toward help-seeking, and psychological well-being.

### Study Hypothesis

- Hypothesis 1: SIGMA (including SIGMA and SIGMA-Booster) and SSIGP are more effective than the active control, ST, in the primary outcome of (1) reducing general anxiety symptoms, and in the secondary outcomes of (2) reducing depressive symptoms, (3) reducing suicidal/self-harming thoughts, (4) reducing hopelessness, (5) enhancing perceived control, (6) increasing positive attitudes toward help-seeking, and (7) enhancing psychological well-being.
- Hypothesis 2: SIGMA (including SIGMA and SIGMA-Booster) is more effective than SSIGP in the outcomes listed from (1) to (7).
- Hypothesis 3: The effectiveness of SIGMA-Booster is greater than that of SIGMA in the outcomes listed from (1) to (7).
- Hypothesis 4: The effectiveness of SIGMA is greater in participants with higher motivation for change than in those with low or no motivation.
- Hypothesis 5: The effectiveness of SIGMA is greater in participants with higher baseline anxiety levels than in those with lower baseline anxiety levels.
- Hypothesis 6: The effectiveness of SIGMA is greater in participants with a more fixed mindset at baseline than in those with a more growth-oriented mindset at baseline.

## Methods

### Ethical Considerations

This study received ethical approval from the Hong Kong Polytechnic University Institutional Review Board (reference number HSEARS20201004001-01) and complied with institutional guidelines and the Declaration of Helsinki. Parental consent and student assent were obtained for all participants. Participants were informed of their right to withdraw at any time without penalty. Data were anonymized (eg, school names removed, unique codes assigned) and stored securely. Participants who completed the entire study received HK $100 (US $13) worth of supermarket coupons as compensation, while participating schools were provided with aggregate mental health reports for institutional support purposes.

### Study Design

The study design was described in the published protocol [27]. The trial was prospectively registered at ClinicalTrials.gov (NCT05027880) under the trial ID NCT05027880.

Unlike the published protocol, we made SIGMA-Booster a separate arm to examine whether booster reminders would help promote the long-term effects of the SSI. Based on the principle of patient and public involvement, we consulted the targeted participant population as well as their teachers and counselors. They suggested that boosters, as reminders, should be helpful for long-term changes. Thus, we designed 5 boosters and printed them into folders to be used as weekly reminders between the 2- and 8-week follow-ups (from week 3 to week 7). Only the SIGMA-Booster group received these boosters.

**Figure 1:**
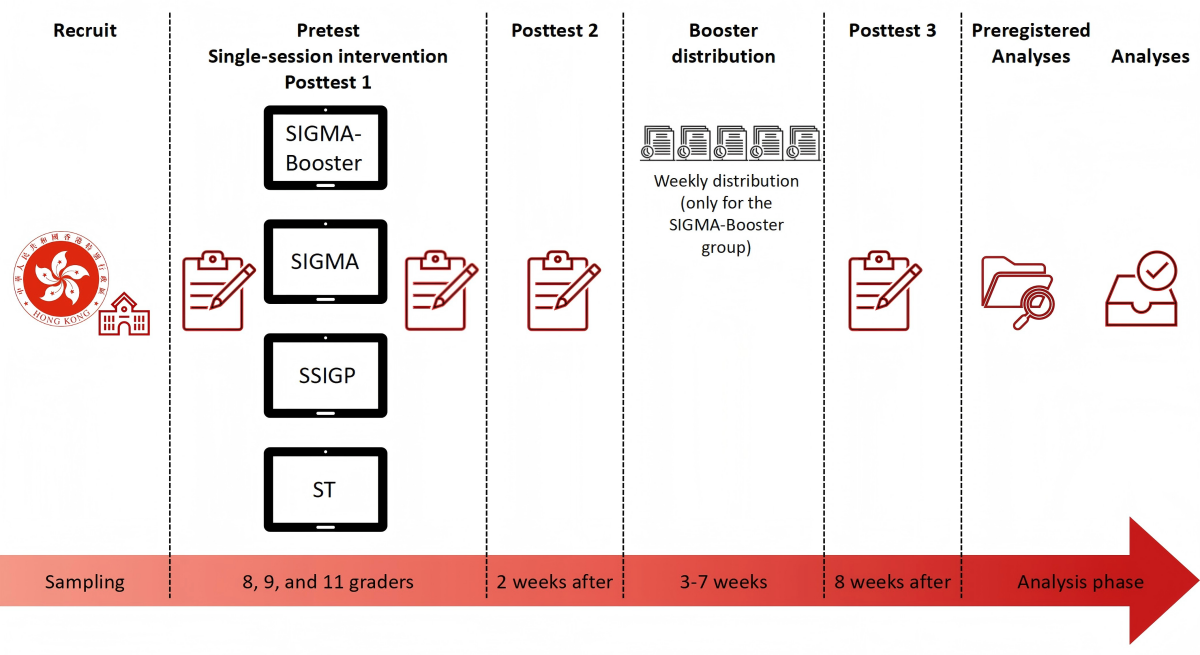
Design of the 4-arm waitlist randomized controlled trial. SIGMA: single-session intervention of a growth mindset for anxiety; SSIGP: single-session intervention of a growth mindset of personality; ST: support therapy.

**Figure 2:**
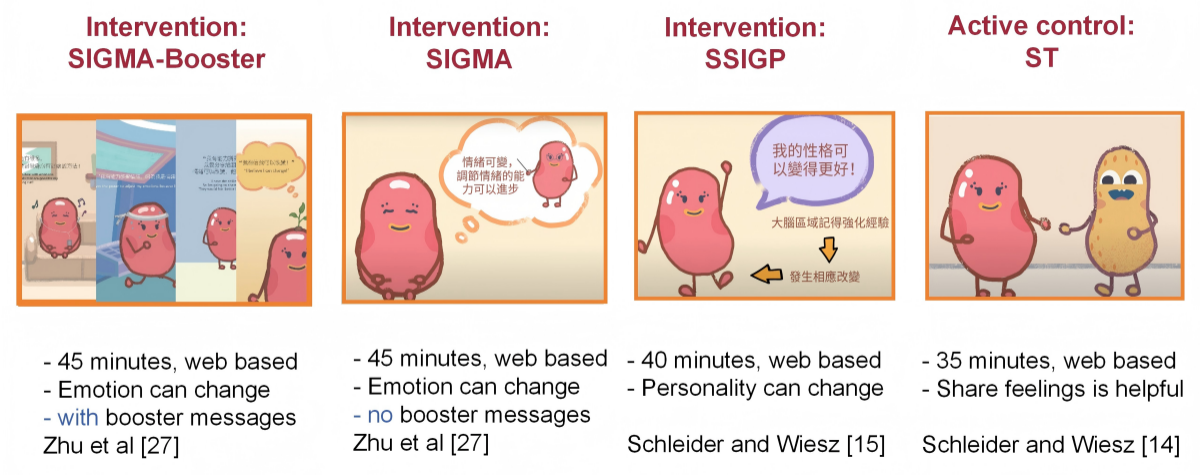
Intervention designs. SIGMA: single-session intervention of a growth mindset for anxiety; SSIGP: single-session intervention of a growth mindset of personality; ST: support therapy.

Thus, 4 classes from the same grade of the participating school were randomized (using computer-generated random numbers) into the (1) SIGMA group, (2) SIGMA-Booster group, (3) SSIGP group, and (4) ST group (active control condition group), all of which received the ST intervention at the same time (Figures 1 and 2). Participants in the SIGMA-Booster arm received the SIGMA intervention along with weekly reminders of key intervention messages as boosters from weeks 3 to 7. Two schools did not have enough classes in 1 grade, so classes from other grades at those schools were invited to join the study. All participants received regular interventions at school. Three repeated assessments were conducted for the 4 groups simultaneously at (1) baseline, (2) 2 weeks postintervention, and (3) 8 weeks postintervention. The cluster randomization at the classroom level helped balance the risk of contamination between the experimental and active control groups, as well as account for school heterogeneity due to factors such as school culture, schedule, and management.

### Participants

Seven schools from Hong Kong Island, Kowloon, and the New Territories participated in the study. These included schools that used English, Chinese, and mixed English and Chinese as the teaching medium. We targeted participants from form 2 to form 3 (grades 8-9). If a school did not have 4 classes in 1 grade, or if the number of eligible participants in a class was too few, we invited additional classes from other grades to participate.

### Inclusion Criteria

Eligible participants were recruited from the 7 secondary schools through cluster randomized sampling. We included participants who (1) were Chinese youth able to read and write Chinese, (2) had sufficient visual and auditory abilities to complete the intervention and assessment, and (3) were able to give assent to participate in the study. As 2 schools did not have enough classes in 1 grade and invited classes from other grades to participate, a few participants above the age of 16 years were included, which did not follow the inclusion criteria outlined in the protocol (12-16 years old).

### Exclusion Criteria

Exclusion criteria included (1) lack of parental consent; (2) inability to stay focused for the duration of the intervention, which is approximately 45 minutes; and (3) intellectual disability or severe illness or pain that could introduce significant bias in the students’ health and mental health conditions. Eligible participants were not screened for anxiety symptoms, so this study comprehensively examined the efficacy of the interventions among students with absent, mild, moderate, and severe levels of anxiety.

### Procedure

The school and student recruitment process included the following steps: First, we sent research invitations to schools randomly selected from the school list. Invitations ceased when 7 schools agreed to participate. To ensure sufficient participant recruitment, we recruited 1 additional school beyond the protocol’s plan. Four classes from the same selected grade(s) at each school were randomly chosen to join the study. The 4 selected classes were then randomized to the SIGMA condition, SIGMA-Booster condition, SSIGP condition, and the active control condition using random numbers. All students in those classes were invited to participate, with final participation contingent upon parental consent and students’ assent.

**Figure 3:**
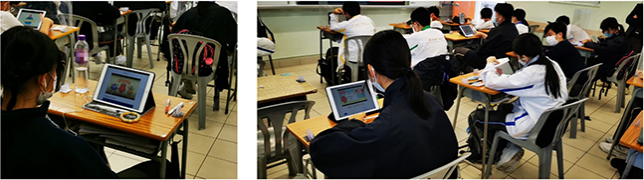
Intervention in a classroom.

After providing consent, students scanned a QR code to access the baseline questionnaire and the intervention program via the Qualtrics survey system (SAP SE). Students within the same class were assigned to the same intervention conditions. The interventions were conducted separately for each group in the school activity rooms, which were equipped with sufficient computers or tablets and headphones (Figure 3). All intervention activities were self-administered and delivered in a web-based format. The principal investigator (SZ) and well-trained research assistants remained in the intervention rooms to provide guidance and assistance if needed. All groups in the same school received interventions concurrently to minimize the influence of the time factor.

### Trial Power and Sample Size

To ensure the sample size was sufficient to test the hypotheses, a small to medium effect size (Cohen *d*=0.33) was used based on prior research [15]. Power was set at 0.80, and α was set at .05. A final sample size of 584 (146 per arm) was required. Considering the attrition rate from our previous studies in school settings (<20%), the baseline recruitment target was set at 732 participants (183 per arm). We ultimately recruited 731 participants. As the number of participants in each class varied, the final number of participants in each of the 4 arms differed.

### Measures

#### Primary Outcome

Anxiety symptoms, measured using the 7-item Generalized Anxiety Disorder (GAD-7) scale [28,29], were the primary outcome. The 7 items assessed whether anxiety symptoms had bothered the individual during the previous 2 weeks, with frequency ranging from 0 (not at all) to 3 (nearly every day). Example items included: “Feeling nervous, anxious, or on edge” and “Not being able to stop or control worrying.” The GAD-7 is a self-rating scale that effectively reflects symptom severity in adolescents and is highly correlated with clinician-administered ratings of anxiety symptoms. It is brief and suitable for self-report studies [30]. The Cronbach α for the scale was 0.93 [31]. By summing the scores of the 7 items, the following classifications were used: 0-4 indicated the absence of anxiety symptoms, 5-9 indicated mild anxiety, 10-14 indicated moderate anxiety, and 15-21 indicated severe anxiety. Based on the severity of anxiety, participants were categorized into 2 groups: the high anxiety group (scores of 10-21) and the nonhigh anxiety group (scores of 0-9) in this study.

#### Secondary Outcomes

##### The 9-Item Patient Health Questionnaire

The 9-item Patient Health Questionnaire (PHQ-9) [32,33] was used to assess participants’ depression levels and suicidal/self-hurting thoughts over the previous 2 weeks, with frequency ranging from 0 (not at all) to 3 (nearly every day). The first 8 items (PHQ-8) were used to measure depression severity (sum of the 8 items), while the last item assessed suicidal/self-hurting thoughts. Responses of 1-3 on the last item were coded as “yes,” and a response of 0 was coded as “no.” Example items from the PHQ-9 included “Little interest or pleasure in doing things” and “Feeling down, depressed, or hopeless.” The item assessing suicidal/self-hurting thoughts was “Thoughts that you would be better off dead or of hurting yourself in some way.”

##### Perceived Control

The Anxiety Control Questionnaire [34] is a 15-item tool that measures participants’ perceived control over their anxiety. The Emotion Control subscale, 1 of the 3 validated subscales, consists of 5 items (eg, “I am able to control my level of anxiety”), including 1 reverse-scored item (When I am anxious, I find it hard to focus on anything other than my anxiety). Responses are rated from 0 (strongly disagree) to 5 (strongly agree). The sum of the 5 items indicates the level of perceived control. The Cronbach α was 0.73.

##### Hopelessness

The 4-item Helplessness subscale of the Demoralization Scale [35] was used to measure participants’ outlook on the future. Each item was rated on a 5-point Likert scale, ranging from 1 (strongly disagree) to 5 (strongly agree). The sum of the 4 items was used to assess hopelessness, with a higher score indicating a correspondingly higher level of hopelessness. An example item was “I feel hopeless.” The Cronbach α for the Chinese version of this Helplessness subscale was 0.72 [36].

##### Attitude Toward Seeking Help

We used 3 items from the Attitude Toward Seeking Counselling Help Assessment [37] to measure participants’ understanding of counseling and attitudes toward seeking counseling help. Example items included “If I believed I was having a mental breakdown, my first inclination would be to get professional attention” and “Professional counseling and treatments can help people improve mental health.” The Cronbach α was 0.72 [37]. Additionally, 2 items were used to assess participants’ intention to seek help. The 2 items were “When I encounter difficulties, I will not ask for help from teachers” and “When I encounter difficulties, I will not ask for help from social workers/counselors.” These 5 items were rated on a 7-point Likert scale, ranging from 1 (strongly disagree) to 7 (strongly agree). After reverse-scoring the 2 help-seeking intention items, the sum score of the 5 items represented participants’ attitudes toward seeking help, with a higher score indicating more positive attitudes toward seeking help.

##### Psychological Well-Being

The short version of the 14-item Warwick-Edinburgh Mental Well-Being Scale (WEMWBS-14) [38,39] was used to measure the extent to which participants generally experienced well-being states. The WEMWBS-14 consists of 14 items, each rated on a 5-point Likert scale ranging from 1 (none of the time) to 5 (all of the time). The sum score of the 14 items indicated the participants’ overall well-being level. An example item was “I have been feeling optimistic about the future.” The scale’s Cronbach α was 0.93.

#### Fidelity Checking and Intervention Feedback

##### Mindsets of Negative Emotions

The validated Chinese version of the 12-item Mindset of Depression, Anxiety, and Stress Scale (MDASS) was used to assess participants’ beliefs regarding the changeability of negative emotional states, such as depression, anxiety, and stress [24]. Sample items included: “When you have a certain level of depression, you really cannot do much to change it,” “To be honest, people cannot really change how anxious they are,” and “No matter how hard people try, they cannot really change the level of stress that they have.” Each item was scored on a 6-point Likert scale ranging from 1 (strongly disagree) to 6 (strongly agree); a higher score indicated a more fixed mindset toward negative emotions (Cronbach α=0.94). It included 3 subscales: depression mindset, anxiety mindset, and stress mindset, with 4 items in each subscale. The Cronbach α values for the 3 subscales were 0.91, 0.89, and 0.90, respectively [24]. By summing the total score, we defined fixed and growth mindsets using a cutoff score of 42, which was calculated by adding the midpoint (ie, 3.5) of the 6-point scale for the 12 items. Individuals scoring equal to or greater than 42 were considered to have more fixed mindsets, while those scoring below this cutoff were considered more inclined to have growth mindsets.

##### Mindset of Personality

Three items from implicit theories of personality [40,41] were used to measure the belief in the changeability of personality on a 6-point Likert scale, ranging from 1 (strongly disagree) to 6 (strongly agree). A higher score indicated a more fixed mindset of personality. A sample item was “People can do things differently, but the important parts of who they are can’t really be changed.” The Cronbach α was 0.85.

##### Motivation to Apply What Was Learned From the Program

In addition to the baseline assessment, immediately after the intervention, participants were asked to rate the extent to which they would like to apply the intervention content and improve their emotion regulation on a 6-point Likert scale (1-6), with a higher score indicating higher motivation. We defined high and low motivation groups based on the median cutoff after summing the 2 motivation items.

##### The Intervention Feedback Scale

This scale was developed based on the Theoretical Framework of Acceptability, which includes 7 component constructs: affective attitude, burden, intervention coherence, perceived effectiveness, opportunity costs, self-efficacy, and ethicality [42]. A general acceptability item, 6 items corresponding to the 6 components of the Theoretical Framework of Acceptability (excluding ethicality), and 4 items—including an open-ended written feedback item—were integrated to comprehensively assess the acceptability of the intervention. These items were drawn from the well-validated Program Feedback Scale [43]. For the Feedback Scale, except for the open-ended item, the other 10 items were assessed on a 5-point scale (eg, “How acceptable was the intervention to you?” with responses ranging from “1=completely unacceptable” to “5=completely acceptable”). The Feedback Scale was administered immediately after the intervention.

##### Attention-Checking Items

To ensure data quality and assess participant attention, 2 attention-checking items were included at all assessment points (baseline and follow-up surveys). These items directly instructed participants to select a specific option based on the given instructions. A sample question was “Please select ‘strongly agree’ for this item.”

#### Sociodemographic Information

Sociodemographic information of participants was collected at baseline to examine group variability in factors such as gender, age, grade, ethnicity, and socioeconomic status (SES).

### Data Analysis

We used an intention-to-treat approach in our primary analysis, where all participants who consented to participate were included. Participants who completed all assessments and passed both attention-checking items at each assessment point were classified in the per-protocol population. A per-protocol analysis was conducted as a sensitivity analysis. Multilevel modeling was used to account for the cluster randomization of classes within the same school [44]. All percentages and scores were presented with 1 decimal place. To examine the effects of the interventions, generalized estimating equations were used to test the group effect, time effect, and their interaction effect on outcome measures. A statistically significant interaction effect indicated the effectiveness of the treatments. Additionally, we calculated effect sizes using estimated marginal means. These effect sizes, expressed as Cohen *d*, compared mean gain scores reflecting changes in each outcome from baseline to the 2 follow-ups for youth receiving the mindset versus active control interventions. The effect sizes were also compared between participants in the intervention group who received booster messages and those who did not. Additionally, we tested the following moderators on the effectiveness of the treatment for the primary and secondary outcomes: baseline anxiety level (dichotomized by the severity of GAD-7), motivation for change (dichotomized by the median of the sum motivation score on the 2 motivation items), and mindset group (fixed vs growth). The corrected quasi-likelihood under the independence model criterion (QICC) for the models with and without the moderator was examined, with evidence of a moderation effect indicated by a smaller QICC for the models including the moderator. Multiple comparisons were not conducted, and a *P* value of <.05 was considered statistically significant. Data analysis was performed using SPSS version 26 (IBM Corp.).

## Results

### Recruitment

**Figure 4:**
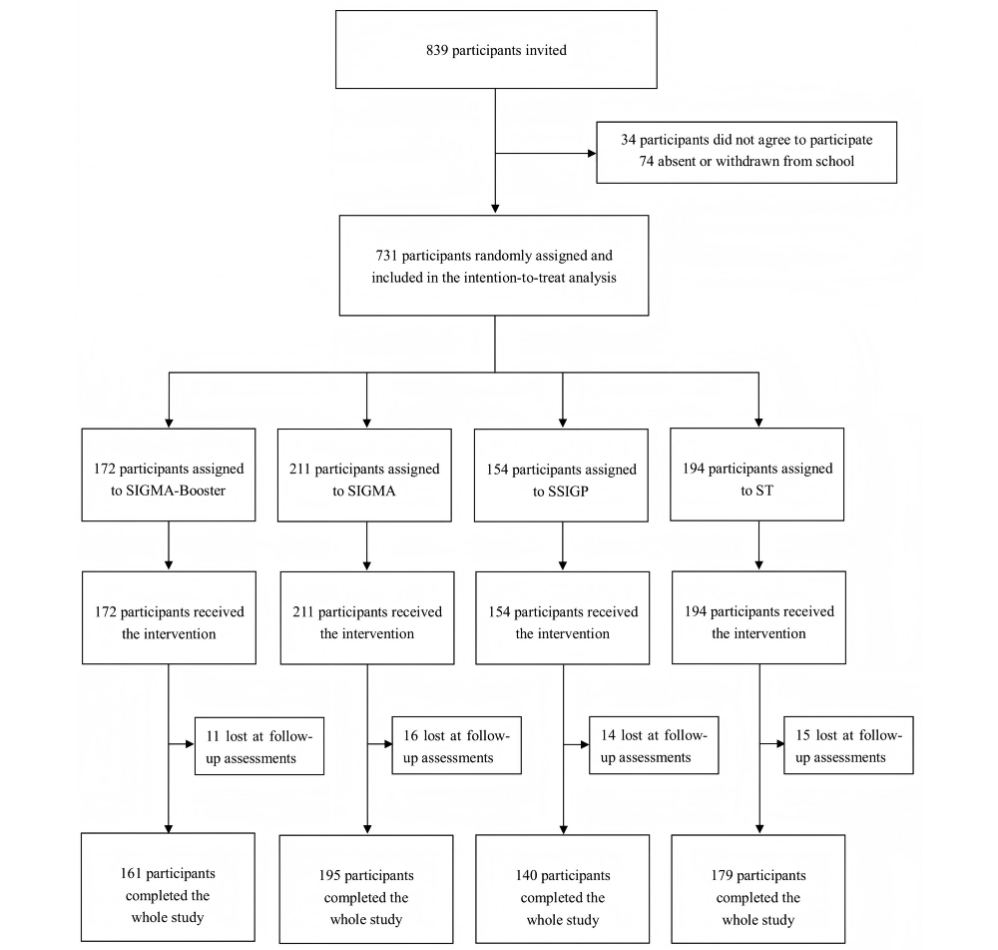
CONSORT (Consolidated Standards of Reporting Trials) flowchart. SIGMA: single-session intervention of a growth mindset for anxiety; SSIGP: single-session intervention of a growth mindset of personality; ST: support therapy.

Figure 4 depicts the CONSORT (Consolidated Standards of Reporting Trials) diagram of the recruitment and participation flow (also see Multimedia Appendix 1). A total of 731 participants were recruited and randomized into 4 groups: SIGMA-Booster (n=172, 23.5%), SIGMA (n=211, 28.9%), SSIGP (n=154, 21.1%), and ST (n=194, 26.5%). All participants received the interventions and were contacted for follow-ups. All participants were included in the intention-to-treat analysis. No participants explicitly requested to be removed from the trial.

### Baseline Characteristics of Participants

Table 1 summarizes the baseline characteristics of the recruited participants. Of the 731 participants, 421 (57.6%) were girls. Statistical differences in grade and age were observed among the 4 groups, as some schools selected classes from 2 different grades. However, there were no statistical differences in ethnicity or SES.

There were no significant differences between the intervention groups on the primary and secondary outcome measures at baseline: anxiety symptoms, *F*_3,727_=0.25, *P*=.86; depressive symptoms, *F*_3,727_=0.13, *P*=.94; suicidal/self-hurting thoughts, *χ*^2^_3_ (N=731)=3.66, *P*=.30; perceived control, *F*_3,727_=0.31, *P*=.82; hopelessness, *F*_3,727_=0.74, *P*=.53; attitude toward seeking help, *F*_3,727_=0.61, *P*=.61; and psychological well-being, *F*_3,727_=0.63, *P*=.60.

**Table 1:** Sample characteristics.

Variables			SIGMA-Booster^a^ (n=172)		SIGMA^b^ (n=211)		SSIGP^c^ (n=154)		ST^d^ (n=194)		Overall (N=731)		*P* value
**Age**													<.001
	Mean (SD)	14.0 (0.9)		14.0 (1.0)		14.5 (1.5)		13.8 (0.8)		14.1 (1.1)			
	Range	12-16		12-18		12-20		12-18		12-20			
	Missing, n (%)	0 (0)		1 (0.5)		0 (0)		0 (0)		1 (0.1)			
**Gender, n (%)**													.005
	Male	55 (32.0)		94 (44.5)		64 (41.6)		97 (50.0)		310 (42.4)			
	Female	117 (68.0)		117 (55.5)		90 (58.4)		97 (50.0)		421 (57.6)			
**Ethnicity, n (%)**													.67
	Chinese	168 (97.7)		204 (96.7)		152 (98.7)		189 (97.4)		713 (97.5)			
	Other	4 (2.3)		7 (3.3)		2 (1.3)		5 (2.6)		18 (2.5)			
**Grade, n (%)**													<.001
	Secondary 2	80 (46.5)		99 (46.9)		59 (38.3)		135 (69.6)		373 (51.0)			
	Secondary 3	92 (53.5)		112 (53.1)		69 (44.8)		59 (30.4)		332 (45.4)			
	Secondary 5	0 (0)		0 (0)		26 (16.9)		0 (0)		26 (3.6)			
**Socioeconomic status, n (%)**													.75
	Low	0 (0.0)		1 (0.5)		1 (0.6)		0 (0)		2 (0.3)			
	Medium	139 (80.8)		172 (81.5)		120 (77.9)		151 (77.8)		582 (79.6)			
	High	33 (19.2)		38 (18.0)		33 (21.4)		43 (22.2)		147 (20.1)			
Willingness to participate in emotional control course (1-6), mean (SD)			3.5 (1.3)		3.5 (1.3)		3.3 (1.3)		3.4 (1.4)		3.4 (1.3)		.69
Willingness to improve emotional control (1-6), mean (SD)			4.2 (1.3)		4.0 (1.2)		4.0 (1.3)		4.0 (1.4)		4.0 (1.3)		.46
Mindset of anxiety (4-24), mean (SD)			14.6 (4.2)		13.8 (4.6)		14.5 (4.6)		13.7 (4.7)		14.1 (4.5)		.17
Mindset of depression (4-24), mean (SD)			13.6 (4.9)		12.8 (5.2)		13.4 (5.0)		12.8 (5.2)		13.1 (5.1)		.32
Mindset of stress (4-24), mean (SD)			15.5 (4.8)		14.3 (5.0)		14.6 (5.2)		14.9 (5.4)		14.8 (5.1)		.14
Mindset of personality (3-18), mean (SD)			13.5 (3.2)		13.0 (3.5)		13.0 (3.2)		13.1 (3.5)		13.1 (3.4)		.51
7-item Generalized Anxiety Disorder (0-21), mean (SD)			6.9 (5.1)		6.6 (5.4)		7.0 (5.1)		6.9 (5.8)		6.8 (5.4)		.86
8-item Patient Health Questionnaire-8 (0-24), mean (SD)			7.3 (5.6)		7.1 (5.7)		7.2 (5.4)		7.4 (5.6)		7.2 (5.6)		.94
Anxiety Control Questionnaire—Emotion Control (0-25), mean (SD)			13.6 (5.0)		13.5 (4.9)		13.7 (4.8)		13.2 (5.4)		13.5 (5.0)		.82
Demoralization Scale—Helplessness (4-20), mean (SD)			9.8 (3.6)		10.2 (4.0)		9.6 (3.8)		9.9 (4.0)		9.9 (3.9)		.53
Attitude Toward Seeking Help (5-35), mean (SD)			19.3 (4.6)		19.3 (5.6)		18.7 (5.2)		19.4 (5.6)		19.2 (5.3)		.61
Warwick-Edinburgh Mental Well-Being Scale (14-70), mean (SD)			41.7 (9.9)		42.3 (11.0)		43.1 (10.8)		43.0 (11.0)		42.5 (10.7)		.60
**Suicidal/self-hurting thoughts, n (%)**													.30
	Yes	53 (30.8)		78 (37.0)		50 (32.5)		55 (28.4)		236 (32.3)			
	No	119 (69.2)		133 (63.0)		104 (67.5)		139 (71.6)		495 (67.7)			

^a^SIGMA-Booster: SIGMA with boosters.

^b^SIGMA: single-session intervention of a growth mindset for anxiety.

^c^SSIGP: single-session intervention of a growth mindset of personality.

^d^ST: support therapy.

### Changes in Primary and Secondary Outcomes

**Figure 5:**
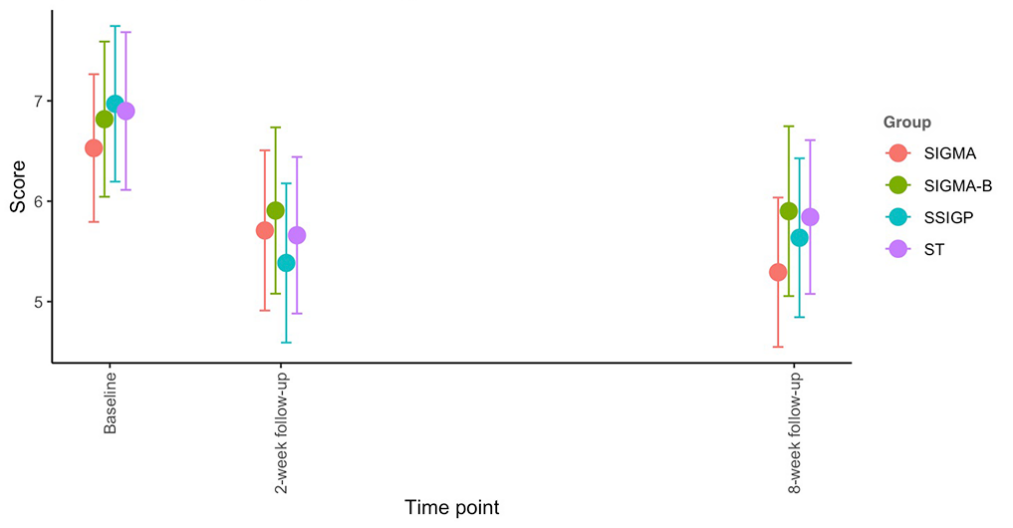
The 7-item Generalized Anxiety Disorder scale (total score) changes over time. Each dot represents the mean score of each group at each time point. Each line around the dot represents the 95% Wald CI of the mean. SIGMA: single-session intervention of a growth mindset for anxiety; SIGMA-B: SIGMA-Booster; SSIGP: single-session intervention of a growth mindset of personality; ST: support therapy.

For the primary outcome, we found a significant main effect of time (*P*<.001), but no significant main effect of group (*P*=.88) or group-by-time interaction (*P*=.54). Participants in all 4 intervention groups showed significant improvement in anxiety symptoms at both the 2- and 8-week follow-ups: *P*_2 weeks_=.02 and *P*_8 weeks_*=*.02 for SIGMA-Booster; *P*_2 weeks_=.006, *P*_8 weeks_<.001 for SIGMA; *P*_2 weeks_<.001, *P*_8 weeks_<.001 for SSIGP, and *P*_2 weeks_<.001, *P*_8 weeks_*=*.003 for ST. This improvement was sustained at the 8-week follow-up, and no significant differences were observed between the 2- and 8-week follow-ups: *P*=.99 for SIGMA-Booster, *P*=.17 for SIGMA, *P*=.46 for SSIGP, and *P=*.59 for ST (Multimedia Appendix 2 and Figure 5). When comparing the changes from baseline to follow-up between each pair of groups, the SSIGP intervention appeared to be more effective than SIGMA (including SIGMA and SIGMA-Booster) in reducing general anxiety symptoms. However, the effect sizes ranged from very small to small (Table 2).

**Table 2:** Effect sizes^a^ of the treatment and intention-to-treat population.

Outcome variables			2-week follow-up, Cohen *d* (SE)		8-week follow-up, Cohen *d* (SE)
**7-item Generalized Anxiety Disorder**					
	SIGMA-Booster^b^ versus SIGMA^c^	–0.02 (0.12)		0.06 (0.12)	
	SIGMA-Booster versus SSIGP^d^	0.13 (0.13)		0.08 (0.13)	
	SIGMA-Booster versus ST^e^	0.06 (0.13)		0.03 (0.13)	
	SIGMA versus SSIGP	0.14 (0.15)		0.02 (0.15)	
	SIGMA versus ST	0.08 (0.15)		–0.03 (0.15)	
	SSIGP versus ST	–0.06 (0.15)		–0.05 (0.15)	
**8-item Patient Health Questionnaire**					
	SIGMA-Booster versus SIGMA	0.06 (0.13)		0.01 (0.13)	
	SIGMA-Booster versus SSIGP	0.10 (0.13)		–0.03 (0.13)	
	SIGMA-Booster versus ST	0.06 (0.13)		–0.04 (0.13)	
	SIGMA versus SSIGP	0.04 (0.15)		–0.04 (0.15)	
	SIGMA versus ST	–0.002 (0.15)		–0.05 (0.15)	
	SSIGP versus ST	–0.05 (0.16)		–0.01 (0.16)	
**Suicidal/self-hurting thoughts**					
	SIGMA-Booster versus SIGMA	0.20 (0.13)		0.18 (0.13)	
	SIGMA-Booster versus SSIGP	0.18 (0.13)		0.27 (0.13)	
	SIGMA-Booster versus ST	0.03 (0.13)		0.04 (0.13)	
	SIGMA versus SSIGP	–0.02 (0.15)		0.09 (0.15)	
	SIGMA versus ST	–0.17 (0.15)		–0.14 (0.15)	
	SSIGP versus ST	–0.15 (0.15)		–0.23 (0.15)	
**Anxiety Control Questionnaire—Emotion Control**					
	SIGMA-Booster versus SIGMA	–0.004 (0.13)		–0.01 (0.13)	
	SIGMA-Booster versus SSIGP	–0.03 (0.13)		0.05 (0.14)	
	SIGMA-Booster versus ST	–0.14 (0.13)		–0.26 (0.13)	
	SIGMA versus SSIGP	–0.03 (0.15)		0.06 (0.15)	
	SIGMA versus ST	–0.13 (0.15)		–0.25 (0.15)	
	SSIGP versus ST	–0.10 (0.15)		–0.31 (0.16)	
**Demoralization Scale—Helplessness**					
	SIGMA-Booster versus SIGMA	0.24 (0.13)		0.16 (0.12)	
	SIGMA-Booster versus SSIGP	0.07 (0.13)		0.05 (0.12)	
	SIGMA-Booster versus ST	0.20 (0.13)		0.08 (0.13)	
	SIGMA versus SSIGP	–0.18 (0.15)		–0.11 (0.15)	
	SIGMA versus ST	–0.04 (0.15)		–0.08 (0.15)	
	SSIGP versus ST	0.14 (0.15)		0.03 (0.15)	
**Attitude Toward Seeking Help**					
	SIGMA-Booster versus SIGMA	–0.03 (0.11)		0.05 (0.11)	
	SIGMA-Booster versus SSIGP	–0.15 (0.11)		–0.05 (0.12)	
	SIGMA-Booster versus ST	–0.12 (0.12)		0.08 (0.12)	
	SIGMA versus SSIGP	–0.12 (0.14)		–0.09 (0.14)	
	SIGMA versus ST	–0.09 (0.14)		0.03 (0.14)	
	SSIGP versus ST	0.04 (0.14)		0.12 (0.15)	
**Warwick-Edinburgh Mental Well-Being Scale**					
	SIGMA-Booster versus SIGMA	0.02 (0.12)		0.02 (0.13)	
	SIGMA-Booster versus SSIGP	0.02 (0.13)		0.10 (0.13)	
	SIGMA-Booster versus ST	–0.08 (0.12)		–0.11 (0.13)	
	SIGMA versus SSIGP	–0.003 (0.16)		0.08 (0.16)	
	SIGMA versus ST	–0.10 (0.15)		–0.13 (0.16)	
	SSIGP versus ST	–0.10 (0.16)		–0.21 (0.15)	

^a^Effect size values were calculated by subtracting the latter group’s mean gain score from the former group’s mean gain score for each outcome from baseline to the 2- and 8-week follow-ups, then dividing by the pooled SD of all participants at baseline.

^b^SIGMA-Booster: SIGMA with boosters.

^c^SIGMA: single-session intervention of a growth mindset for anxiety.

^d^SSIGP: single-session intervention of a growth mindset of personality.

^e^ST: support therapy.

**Figure 6:**
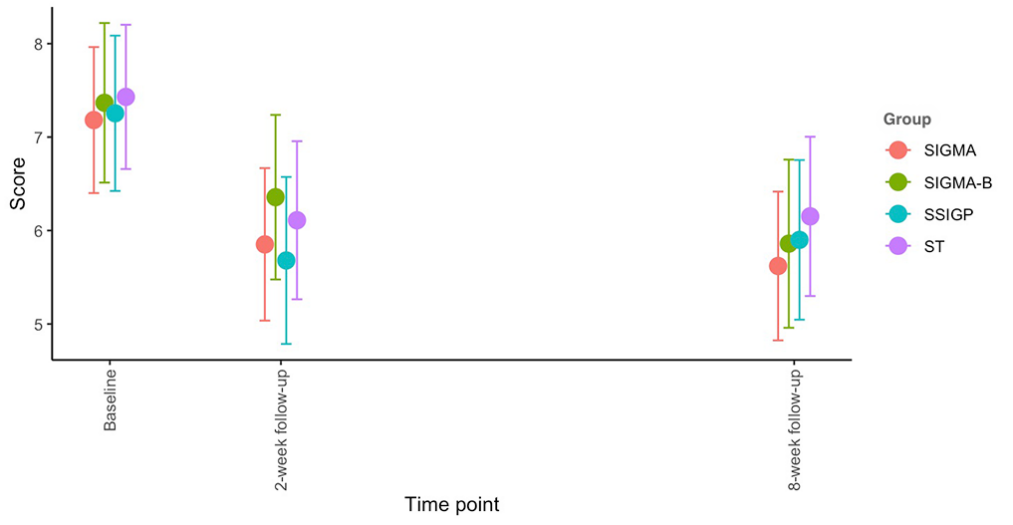
The 8-item Patient Health Questionnaire scale (total score) changes over time. Each dot represents the mean score of each group at each time point. Each line around the dot represents the 95% Wald CI of the mean. SIGMA: single-session intervention of a growth mindset for anxiety; SIGMA-B: SIGMA-Booster; SSIGP: single-session intervention of a growth mindset of personality; ST: support therapy.

**Figure 7:**
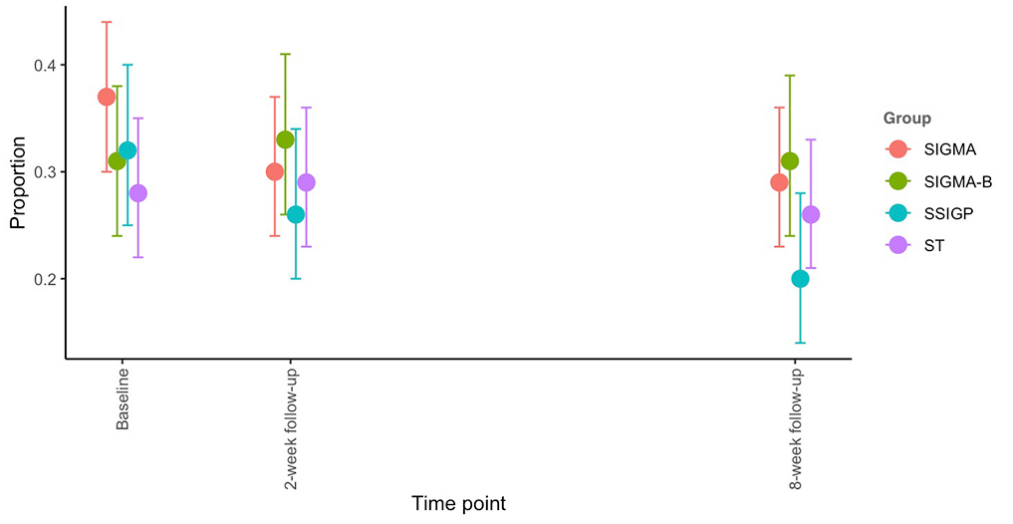
The proportion of participants with suicidal/self-hurting thoughts changes over time. Each dot represents the mean score of each group at each time point. Each line around the dot represents the 95% Wald CI of the mean. SIGMA: single-session intervention of a growth mindset for anxiety; SIGMA-B: SIGMA-Booster; SSIGP: single-session intervention of a growth mindset of personality; ST: support therapy.

For the secondary outcomes, all the main effects of time were significant, while the main effects of group and the interaction effects of group and time were insignificant (main effects of group: *P=*.87 for depressive symptoms; *P*=.41 for suicidal/self-hurting thoughts; *P*=.97 for perceived control; *P*=.73 for hopelessness; *P*=.87 for help-seeking attitude; *P*=.39 for psychological well-being and interaction effects; *P=*.85 for depressive symptoms; *P*=.10 for suicidal/self-hurting thoughts; *P*=.20 for perceived control; *P*=.23 for hopelessness; *P*=.33 for help-seeking attitude; and *P*=.68 for psychological well-being), similar to the primary outcome. Specifically, the results for depressive symptoms mirrored those for anxiety (main effect of time, *P*<.001 and main effect of group, *P*=.87). All 4 groups showed a reduction in depressive symptoms at follow-ups, and the effects observed at the 8-week follow-up were comparable to those at the 2-week follow-up (see Multimedia Appendix 2 and Figure 6). Moreover, the effect sizes for the comparison between groups on the changes from baseline to follow-ups were all very small (Table 2). Second, a significant main effect of time was found for suicidal/self-hurting thoughts (*P*=.005), but the main effect of group was not significant (*P*=.41). Specifically, the SIGMA group showed a reduction in suicidal/self-hurting thoughts at both the 2- and 8-week follow-ups, with the effect sustained at the 8-week follow-up compared with the 2-week follow-up. The SSIGP group showed a significant reduction in suicidal/self-hurting thoughts at the 8-week follow-up (*P*<.001), but not at the 2-week follow-up (*P*=.14). The other 2 groups did not report significant changes in suicidal/self-hurting thoughts at either follow-up with *P*_2 weeks_=.50, *P*_8 weeks_=.96 for SIGMA-Booster and *P*_2 weeks_=.75, *P*_8 weeks_=.61 for ST (see Multimedia Appendix 2 and Figure 7). Both the SIGMA and SSIGP interventions appeared to be more effective in reducing suicidal/self-hurting thoughts than the SIGMA-Booster and ST, although the effect sizes were small (Table 2). For the other secondary outcomes, the main effects of time were significant for all variables (for perceived control, *P*=.03; for hopelessness, *P*<.001; for help-seeking attitude, *P*<.001; and for psychological well-being, *P*<.001). However, the main effects of the group were not significant (*P* values ranged from .39 to .97). For perceived control, only the ST group reported significant improvement at both the 2- and 8-week follow-ups (*P*_2 weeks_=.02, *P*_8 weeks_=.007). The ST intervention appeared to be more effective than the other 3 groups in improving perceived control, though the effect sizes were small (Table 2). For hopelessness, both the SIGMA and ST groups showed significant improvement at both the 2-week (*P*<.001 for SIGMA and *P*<.001 for ST) and 8-week follow-ups (*P*=.001 for SIGMA and *P*=.005 for ST), while the SSIGP group only reported significant improvement at the 8-week follow-up (*P=*.02). The SIGMA group seemed to outperform the SIGMA-Booster group in reducing hopelessness, although the effect sizes were small (Table 2). For help-seeking attitude, both the SIGMA-Booster and SSIGP groups reported significant improvement at both the 2-week (*P*=.01 for SIGMA-Booster and *P*<.001 for SSIGP) and 8-week follow-ups (*P*=.01 for SIGMA-Booster and *P*=.003 for SSIGP), while the SIGMA and ST groups showed significant improvement only at the 2-week follow-up (*P*=.006 for SIGMA and *P*<.001 for ST). For psychological well-being, the ST group showed significant improvement at both the 2- and 8-week follow-ups with *P*=.01 and *P*<.001, respectively, while the SIGMA-Booster and SIGMA groups demonstrated significant improvement only at the 8-week follow-up with *P*=.02 (Multimedia Appendix 2). The effect sizes for changes in attitude toward seeking help and psychological well-being were all very small between groups. The only effect size that reached a small magnitude (>0.2) was for the improvement in psychological well-being at the 8-week follow-up, where the ST group showed greater improvement compared with the SSIGP group (Table 2).

The sensitivity analysis conducted for the per-protocol population showed results similar to those of the intention-to-treat population. The specific results of the sensitivity analysis can be found in Multimedia Appendix 3.

### Moderation Analysis

We conducted moderation analyses based on baseline anxiety (high/low), motivation to change (high/low), and mindset (growth mindset vs fixed mindset). Including these moderation factors improved model fit, as indicated by the decreases in QICC, ranging from 210 to 31,519.23. This was true for all outcomes, except for suicidal/self-hurting thoughts when motivation level was used as the moderator. These results indicate that baseline anxiety level and mindset had moderating effects on all outcome measures, while motivation level moderated all outcomes except for suicidal/self-hurting thoughts. Participants with higher baseline anxiety, greater motivation to change their situation, and a more fixed baseline mindset showed greater improvements in the outcome measures (see Multimedia Appendices 4-6 for details).

### Intervention Feedback

Participants’ feedback on the SSIs is detailed in Table 3. Most participants reported understanding (536/731, 73.3%) and agreeing (504/731, 68.9%) with the intervention content. More than half found the intervention helpful (465/731, 63.6%) and interesting (417/731, 57.0%). Some indicated that they liked the course (404/731, 55.2%) and expressed a willingness to recommend it to others (402/731, 55.9%). Additionally, 437 out of 731 (59.8%) reported increased confidence in their ability to cope with emotions following the intervention. Meanwhile, very few found this course burdensome (28/731, 3.8%) or interfering (70/731, 9.58%). The findings showed that 459 out of 731 (62.8%) participants indicated that they accept or highly accept the intervention. However, about 237 (32.4%) participants were neutral, indicating that there is still significant room for improvement.

**Table 3:** Feedback on the intervention.

Variables	SIGMA-Booster^a^ (n=172), n (%)	SIGMA^b^ (n=211), n (%)	SSIGP^c^ (n=154), n (%)	ST^d^ (n=194), n (%)	Overall (N=731), n (%)
Like this course	93 (54.1)	113 (53.6)	87 (56.5)	111 (57.2)	404 (55.3)
Understand this course	124 (72.1)	149 (70.6)	116 (75.3)	147 (75.8)	536 (73.3)
This course is useful	104 (60.5)	128 (60.7)	102 (66.2)	131 (67.5)	465 (63.6)
Recommend this course to others	92 (53.5)	117 (55.5)	81 (52.6)	112 (57.7)	402 (55.0)
This course is interesting	91 (52.9)	113 (53.6)	88 (57.1)	125 (64.4)	417 (57.0)
Agree with this course	115 (66.9)	143 (67.8)	106 (68.8)	140 (72.2)	504 (68.9)
Emotional control improved after this course	100 (58.1)	128 (60.7)	85 (55.2)	124 (63.9)	437 (59.8)
Burden in joining this course	9 (5.2)	6 (2.8)	5 (3.2)	8 (4.1)	28 (3.8)
Affect other arrangement due to joining this course	20 (11.6)	15 (7.1)	16 (10.4)	19 (9.8)	70 (9.6)
Acceptance to this course	103 (59.9)	127 (60.2)	110 (71.4)	119 (61.3)	459 (62.8)

^a^SIGMA-Booster: SIGMA with boosters.

^b^SIGMA: single-session intervention of a growth mindset for anxiety.

^c^SSIGP: single-session intervention of a growth mindset of personality.

^d^ST: support therapy.

## Discussion

### Overview

Contrary to our hypotheses, we found that all 4 SSIs, including ST, significantly reduced general anxiety symptoms and improved some secondary outcomes. Although the core messages delivered in approximately 40 minutes differed among these interventions, nearly all improvements in the 4 groups were sustained from the 2-week to the 8-week follow-ups. These findings provide evidence for the effectiveness of low-dosage nonpharmacological interventions in improving youth mental health outcomes. SIGMA, which enhances the belief in change regarding negative emotions, showed an intervention effect on all outcome measures except perceived control. The SSIGP, targeting personality mindset, was more effective in reducing self-harm and suicidal thoughts. ST had a greater effect on perceived control. Surprisingly, the SIGMA-Booster group, which received booster messages, did not achieve better results than the SIGMA group. By contrast, the SIGMA group outperformed the SIGMA-Booster group in outcomes such as reducing suicidal and self-harming thoughts as well as hopelessness. The SIGMA interventions were not more effective than SSIGP, particularly in the primary outcome of reducing anxiety symptoms. Moreover, the ST intervention was especially effective in improving perceived control, outperforming the other 3 interventions. Moderation tests revealed that some adolescents benefited more from the interventions. Consistent with our hypotheses on moderation effects, participants showed greater improvement if they had more severe anxiety symptoms, stronger baseline fixed mindsets, and a greater motivation to change. Although the effect sizes for group comparisons on outcome changes were small, it is encouraging that SSIs produced sustained outcomes over 8 weeks.

Our findings align closely with existing research on SSIs for youth psychiatric problems. A previous meta-analysis by Schleider and Weisz [14] found that a young person receiving an SSI had a 58% likelihood of performing better than a youth in the control group. The effect sizes varied depending on the control conditions, with larger effect sizes observed in studies with no-treatment or waitlist controls (0.41) compared with those with active controls (0.14). In our study, the effect sizes for SIGMA versus SSIGP at the 2-week follow-up were approximately 0.14 (SIGMA vs SSIGP: 0.14 and SIGMA-Booster vs SSIGP: 0.13), while the effect sizes for SIGMA versus ST were smaller. The finding that SSIs were effective for multiple outcomes, including anxiety and depression symptoms, aligns with a recent umbrella review on SSIs, which showed that over 80% of reviews reported significant positive effects on at least one outcome [21]. In summary, our study provides additional evidence supporting the modest yet significant clinical utility of certain SSIs for youth, including those targeting anxiety symptoms [21].

The design of the interventions and the implementation of the RCT for SSIs provided valuable insights and implications for local practice. Generally, these SSIs were designed following recommended guidelines to ensure the efficacy of brief interventions [45-47]. First, we formed a youth advisory group to ensure the content was relevant to the target users. Second, we incorporated cartoons and animated videos into the intervention. The cartoon heroine, “Hong Dou” (the Red Bean), and her friends were created by artists and digital designers to make the animations and videos more engaging. As a result, the intervention videos effectively captured participants’ attention. Third, because SSIs should be highly focused on delivering 1 or a few core messages, each SSI in our study conveyed a single core message to participants. We reinforced learning through multiple methods, including examples, testimonials, authoritative research findings, and saying-is-believing exercises. Fourth, to ensure active participation in the online web-based intervention, we embedded it in the Qualtrics survey tool and included interactive exercises after each session. Participants received timely feedback on these exercises. Feedback from participants indicated that the SSIs in our study were well-suited to adolescents’ needs and expectations for mental health care. This study provides a clear protocol for implementation, including content and strategies, which will be valuable for the future use and development of SSIs.

This study is a pioneering investigation into web-based SSIs among Chinese adolescents. The efficacy of SSIs, particularly self-administered web-based interventions, has been understudied. This study examined the effects of SSIs on mental health among Chinese adolescents using a cluster RCT design. The 4 conditions in this study showed improvements in mental health over 2 weeks, with the effects sustained after 8 weeks. There were no significant group differences among the 4 conditions. As the interventions were implemented at different times across the academic year in different schools, any potential school schedule effects were minimized. On the one hand, these results suggest that providing low-dosage self-help interventions may help adolescents gain insights and strategies for managing their emotions and coping. On the other hand, an RCT with an added waitlist control group would be valuable for further testing the effectiveness of the intervention.

The SIGMA-Booster group did not show a better effect at the 8-week follow-up. There may be several reasons for this. First, there may be no difference between the booster and nonbooster groups. If an SSI has instilled changes in the emotional mindset, those changes could be long-lasting. Second, the booster group might have a greater effect in the longer term, so a more extended follow-up would be necessary to capture any differences between the booster and nonbooster groups. Third, the booster design in this study may not have been effective. Future studies should carefully examine the format and content of booster interventions.

This study makes several significant contributions. First, the SSIs developed and tested with an RCT could serve as an alternative mental health service for adolescents. Although the effect size of the SSI was small, it can benefit a proportion of youth who would otherwise go without services. It can also support youth on the waitlist for psychiatric services by fostering intrinsic motivation and reducing hesitation to seek treatment. It can also complement multisession psychosocial treatments [21]. Second, these SSIs expanded existing mindset interventions to include emotional mindset and adapted them to the Chinese context. This project may provide a generalizable model for the development and implementation of SSIs for youth in the Chinese context. Third, this study initiated the development and evaluation of boosters for SSIs. Although we did not find significant differences among the groups, the findings of this study could serve as a foundation for further research. In summary, the easy-access self-help program enables adolescents with anxiety symptoms to receive timely help and may help reduce the risk of worsening anxiety symptoms and the development of comorbid mental health issues before they can access therapy from a trained therapist or psychiatrist.

### Limitations

There are limitations to consider. First, because this study did not exclude individuals based on the severity of their anxiety symptoms, the efficacy of the interventions in reducing anxiety symptoms among students without anxiety or with very low levels of anxiety may not have been significant, potentially affecting the overall statistical significance. However, future RCT studies could examine the differentiated impacts on youth across a broader range of anxiety problems through subgroup analyses. In this study, we simply divided participants into 2 groups (high baseline anxiety and low baseline anxiety) and examined the moderation effects. Second, there was no waitlist control group, as all groups received specific interventions, with even the control group receiving ST (active control). Adding a waitlist control group would help provide a better understanding of the overall effect of SSIs. Third, although we used cluster randomization at the classroom level to balance the risk of contamination between groups and school heterogeneity, and employed multilevel modeling to account for the clustering of classes within the same school, it was still challenging to completely eliminate the risk of contamination. Students in different classes may interact and share information about their interventions, which could potentially influence the outcomes. Fourth, the study only included a follow-up period of 8 weeks, which may not have allowed us to capture the long-term effects. Future studies with a longer follow-up period will be necessary to better understand the sustained impact of the interventions.

### Conclusions

This study presents evidence-based implementation of web-based single-session growth mindset interventions for adolescent anxiety and compares the efficacy of SSIs using growth mindsets on negative emotions and personality, along with an active control group. The findings support that the easy-access self-help program led to improvements in adolescent anxiety, depression, and suicidal and self-harm thoughts at the 8-week follow-up. These interventions may enable adolescents with anxiety symptoms to access timely help, reducing the risk of worsening anxiety symptoms and the development of comorbid mental health issues before they can access therapy from a trained therapist or psychiatrist. This study also provides an example of implementing SSIs among Chinese adolescents and will contribute to the development of easy-access, low-cost, and scalable interventions for mental health promotion among young people.

## Appendix

Multimedia Appendix 1 CONSORT-eHEALTH checklist (V 1.6.1).

Multimedia Appendix 2 Generalized estimating equation results for the intention-to-treat population.

Multimedia Appendix 3 Generalized estimating equation results for the per-protocol population—presented as estimated marginal means (SE).

Multimedia Appendix 4 Moderating effect of baseline anxiety levels on treatment outcomes.

Multimedia Appendix 5 Moderating effect of baseline motivation levels on treatment outcomes.

Multimedia Appendix 6 Moderating effect of baseline growth mindset levels on treatment outcomes.

## Abbreviations

CONSORT: Consolidated Standards of Reporting Trials
GAD-7: 7-item Generalized Anxiety Disorder
MDASS: Mindset of Depression, Anxiety, and Stress Scale
PHQ-9: 9-item Patient Health Questionnaire
QICC: Corrected Quasi-Likelihood under the Independence Model Criterion
SES: socioeconomic status
SIGMA: single-session intervention of a growth mindset for anxiety
SSI: single-session intervention
SSIGP: single-session intervention of a growth mindset of personality
WEMWBS-14: 14-item Warwick-Edinburgh Mental Well-Being Scale

## Data Availability

Data are not publicly available but can be obtained for proper purposes by contacting the corresponding author.
