# Supplementary material for: Effects of Web-Based Single-Session Growth Mindset Interventions for Reducing Adolescent Anxiety: Four-Armed Randomized Controlled Trial
Source: JMIR Pediatr Parent. 2025 Apr 18;8:e63500. doi: 10.2196/63500 (PMC12048788; doi:10.2196/63500)
Supplement: Multimedia Appendix 2 [file pediatrics_v8i1e63500_app2.docx]

| Outcome variables | | Baseline | 2-week follow-up | 8-week follow-up | *P* value (baseline vs 2-week follow-up) | *P* value (baseline vs 8-week follow-up) | *P* value (2-week vs 8-week follow-up) |
| --- | --- | --- | --- | --- | --- | --- | --- |
| **7-item Generalized Anxiety Disorder** | |  |  |  |  |  |  |
|  | SIGMA-Booster^a^, estimated marginal means (SE) | 6.8 (0.4) | 5.9 (0.4) | 5.9 (0.4) | .02 | .02 | .99 |
|  | SIGMA^b^, estimated marginal means (SE) | 6.5 (0.4) | 5.7 (0.4) | 5.3 (0.4) | .006 | <.001 | .17 |
|  | SSIGP^c^, estimated marginal means (SE) | 7.0 (0.4) | 5.4 (0.4) | 5.6 (0.4) | <.001 | <.001 | .46 |
|  | ST^d^, estimated marginal means (SE) | 6.9 (0.4) | 5.7 (0.4) | 5.8 (0.4) | <.001 | .003 | .59 |
|  | *P*_SIGMA-Booster vs SIGMA_ | .59 | .73 | .28 | *P* value (interaction) | .54 | N/A |
|  | *P*_SIGMA-Booster vs SSIGP_ | .78 | .36 | .65 | N/A | N/A | N/A |
|  | *P*_SIGMA-Booster vs ST_ | .89 | .67 | .92 | N/A | N/A | N/A |
|  | *P*_SIGMA vs SSIGP_ | .41 | .57 | .53 | N/A | N/A | N/A |
|  | *P*_SIGMA vs ST_ | .50 | .93 | .32 | N/A | N/A | N/A |
|  | *P* _SSIGP vs ST_ | .90 | .63 | .72 | N/A | N/A | N/A |
| **8-item Patient Health Questionnaire** | |  |  |  |  |  |  |
|  | SIGMA-Booster, estimated marginal means (SE) | 7.4 (0.4) | 6.4 (0.4) | 5.9 (0.5) | .01 | <.001 | .14 |
|  | SIGMA, estimated marginal means (SE) | 7.2 (0.4) | 5.9 (0.4) | 5.6 (0.4) | <.001 | <.001 | .49 |
|  | SSIGP, estimated marginal means (SE) | 7.3 (0.4) | 5.7 (0.5) | 5.9 (0.4) | <.001 | <.001 | .53 |
|  | ST, estimated marginal means (SE) | 7.4 (0.4) | 6.1 (0.4) | 6.2 (0.4) | <.001 | .001 | .91 |
|  | *P*_SIGMA-Booster vs SIGMA_ | .75 | .40 | .69 | *P* value (interaction) | .85 | N/A |
|  | *P*_SIGMA-Booster vs SSIGP_ | .85 | .28 | .95 | N/A | N/A | N/A |
|  | *P*_SIGMA-Booster vs ST_ | .91 | .69 | .65 | N/A | N/A | N/A |
|  | *P*_SIGMA vs SSIGP_ | .90 | .78 | .63 | N/A | N/A | N/A |
|  | *P*_SIGMA vs ST_ | .66 | .67 | .37 | N/A | N/A | N/A |
|  | *P* _SSIGP vs ST_ | .76 | .49 | .68 |  |  |  |
| **Suicidal/self-hurting thoughts** | |  |  |  |  |  |  |
|  | SIGMA-B, estimated marginal means (SE) | 0.3 (0.04) | 0.3 (0.04) | 0.3 (0.04) | .50 | .96 | .54 |
|  | SIGMA, estimated marginal means (SE) | 0.4 (0.04) | 0.3 (0.03) | 0.3 (0.03) | .04 | .02 | .76 |
|  | SSIGP, estimated marginal means (SE) | 0.3 (0.04) | 0.3 (0.04) | 0.2 (0.03) | .14 | <.001 | .07 |
|  | ST, estimated marginal means (SE) | 0.3 (0.03) | 0.3 (0.03) | 0.3 (0.03) | .75 | .61 | .39 |
|  | *P*_SIGMA-Booster vs SIGMA_ | .21 | .51 | .68 | *P* value (interaction) | .10 | N/A |
|  | *P*_SIGMA-Booster vs SSIGP_ | .76 | .19 | .03 | N/A | N/A | N/A |
|  | *P*_SIGMA-Booster vs ST_ | .58 | .43 | .36 | N/A | N/A | N/A |
|  | *P*_SIGMA vs SSIGP_ | .38 | .47 | .06 | N/A | N/A | N/A |
|  | *P*_SIGMA vs ST_ | .06 | .88 | .59 | N/A | N/A | N/A |
|  | *P* _SSIGP vs ST_ | .40 | .57 | .18 |  |  |  |
| **Anxiety Control Questionnaire—Emotion Control** | |  |  |  |  |  |  |
|  | SIGMA-Booster, estimated marginal means (SE) | 13.7 (0.4) | 14.0 (0.3) | 13.6 (0.3) | .53 | .68 | .22 |
|  | SIGMA, estimated marginal means (SE) | 13.6 (0.3) | 13.9 (0.4) | 13.5 (0.4) | .47 | .78 | .32 |
|  | SSIGP, estimated marginal means (SE) | 13.7 (0.4) | 14.2 (0.4) | 13.3 (0.4) | .20 | .31 | .04 |
|  | ST, estimated marginal means (SE) | 13.2 (0.4) | 14.1 (0.4) | 14.3 (0.4) | .02 | .007 | .68 |
|  | *P*_SIGMA-Booster vs SIGMA_ | .85 | .88 | .94 | *P* value (interaction) | .20 | N/A |
|  | *P*_SIGMA-Booster vs SSIGP_ | .98 | .72 | .65 | N/A | N/A | N/A |
|  | *P*_SIGMA-Booster vs ST_ | .32 | .77 | .15 | N/A | N/A | N/A |
|  | *P*_SIGMA vs SSIGP_ | .83 | .63 | .71 | N/A | N/A | N/A |
|  | *P*_SIGMA vs ST_ | .39 | .67 | .15 | N/A | N/A | N/A |
|  | *P* _SSIGP vs ST_ | .31 | .95 | .08 | N/A | N/A | N/A |
| **Demoralization Scale—Helplessness** | |  |  |  |  |  |  |
|  | SIGMA-Booster, estimated marginal means (SE) | 9.7 (0.3) | 9.6 (0.3) | 9.4 (0.3) | .55 | .13 | .35 |
|  | SIGMA, estimated marginal means (SE) | 10.1 (0.3) | 9.0 (0.3) | 9.1 (0.3) | <.001 | .001 | .79 |
|  | SSIGP, estimated marginal means (SE) | 9.5 (0.3) | 9.1 (0.3) | 8.9 (0.3) | .14 | .02 | .52 |
|  | ST, estimated marginal means (SE) | 9.9 (0.3) | 8.9 (0.3) | 9.1 (0.3) | <.001 | .005 | .37 |
|  | *P*_SIGMA-Booster vs SIGMA_ | .35 | .17 | .52 | *P* value (interaction) | .23 | N/A |
|  | *P*_SIGMA-Booster vs SSIGP_ | .56 | .25 | .28 | N/A | N/A | N/A |
|  | *P*_SIGMA-Booster vs ST_ | .77 | .12 | .64 | N/A | N/A | N/A |
|  | *P*_SIGMA vs SSIGP_ | .14 | .84 | .67 | N/A | N/A | N/A |
|  | *P*_SIGMA vs ST_ | .54 | .83 | .89 | N/A | N/A | N/A |
|  | *P* _SSIGP vs ST_ | .40 | .68 | .59 | N/A | N/A | N/A |
| **Attitude Toward Seeking Help** | |  |  |  |  |  |  |
|  | SIGMA-Booster, estimated marginal means (SE) | 19.3 (0.4) | 20.0 (0.4) | 20.2 (0.4) | .01 | .01 | .73 |
|  | SIGMA, estimated marginal means (SE) | 19.3 (0.4) | 20.3 (0.4) | 20.0 (0.4) | .006 | .06 | .27 |
|  | SSIGP, estimated marginal means (SE) | 18.7 (0.4) | 20.3 (0.4) | 19.8 (0.4) | <.001 | .003 | .17 |
|  | ST, estimated marginal means (SE) | 19.4 (0.4) | 20.8 (0.4) | 19.9 (0.4) | <.001 | .26 | .01 |
|  | *P*_SIGMA-Booster vs SIGMA_ | .87 | .63 | .73 | *P* value (interaction) | .33 | N/A |
|  | *P*_SIGMA-Booster vs SSIGP_ | .31 | .61 | .55 | N/A | N/A | N/A |
|  | *P*_SIGMA-Booster vs ST_ | .81 | .15 | .60 | N/A | N/A | N/A |
|  | *P*_SIGMA vs SSIGP_ | .27 | .97 | .79 | N/A | N/A | N/A |
|  | *P*_SIGMA vs ST_ | .94 | .34 | .83 | N/A | N/A | N/A |
|  | *P* _SSIGP vs ST_ | .24 | .37 | .97 | N/A | N/A | N/A |
| **Warwick-Edinburgh Mental Well-Being Scale** | |  |  |  |  |  |  |
|  | SIGMA-Booster, estimated marginal means (SE) | 42.0 (0.8) | 43.1 (0.8) | 44.2 (0.8) | .08 | .004 | .15 |
|  | SIGMA, estimated marginal means (SE) | 42.7 (0.8) | 43.6 (0.9) | 44.7 (1.0) | .19 | .02 | .15 |
|  | SSIGP, estimated marginal means (SE) | 43.2 (0.9) | 44.1 (1.0) | 44.4 (0.9) | .29 | .18 | .80 |
|  | ST, estimated marginal means (SE) | 43.0 (0.8) | 45.0 (0.9) | 46.4 (0.9) | .01 | <.001 | .04 |
|  | *P*_SIGMA-Booster vs SIGMA_ | .49 | .68 | .67 | *P* value (interaction) | .68 | N/A |
|  | *P*_SIGMA-Booster vs SSIGP_ | .28 | .42 | .87 | N/A | N/A | N/A |
|  | *P*_SIGMA-Booster vs ST_ | .34 | .12 | .06 | N/A | N/A | N/A |
|  | *P*_SIGMA vs SSIGP_ | .68 | .70 | .80 | N/A | N/A | N/A |
|  | *P*_SIGMA vs ST_ | .78 | .27 | .19 | N/A | N/A | N/A |
|  | *P* _SSIGP vs ST_ | .89 | .49 | .11 | N/A | N/A | N/A |

^a^SIGMA-Booster: SIGMA with boosters.

^b^SIGMA: single-session intervention of growth mindset for anxiety.

^c^SSIGP: single-session intervention of growth mindset of personality.

^d^ST: support therapy.
