# Supplementary material for: Effects of Web-Based Single-Session Growth Mindset Interventions for Reducing Adolescent Anxiety: Four-Armed Randomized Controlled Trial
Source: JMIR Pediatr Parent. 2025 Apr 18;8:e63500. doi: 10.2196/63500 (PMC12048788; doi:10.2196/63500)
Supplement: Multimedia Appendix 3 [file pediatrics_v8i1e63500_app3.docx]

**Multimedia Appendix 3.** Generalised estimating equation results, per-protocol population (estimated marginal means (standard error))

| Outcome variables | Baseline | 2-week follow-up | 8-week follow-up | *P*-value (Baseline  vs 2-week follow-up) | *P*-value (Baseline vs 8-week follow-up) | *P*-value  (2-week  vs 8-week follow-up) |
| --- | --- | --- | --- | --- | --- | --- |
| 7-item Generalized Anxiety Disorder |  |  |  |  |  |  |
| SIGMA-Booster^a^, estimated marginal means (SE) | 7.2 (0.7) | 5.7 (0.6) | 6.9 (0.7) | .01 | .69 | .02 |
| SIGMA^b^, estimated marginal means (SE) | 6.6 (0.7) | 5.5 (0.8) | 5.7 (0.8) | .008 | .13 | .72 |
| SSIGP^c^, estimated marginal means (SE) | 6.9 (0.7) | 5.7 (0.7) | 5.9 (0.7) | .02 | .04 | .77 |
| ST^d^, estimated marginal means (SE) | 6.5 (0.6) | 5.2 (0.6) | 5.7 (0.6) | .001 | .07 | .15 |
| *P*_SIGMA-B vs SIGMA_ | .55 | .84 | .25 | *P*-value (interaction) | .84 | N/A |
| *P*_SIGMA-B vs SSIGP_ | .82 | .99 | .30 | N/A | N/A | N/A |
| *P*_SIGMA-B vs ST_ | .44 | .54 | .19 | N/A | N/A | N/A |
| *P*_SIGMA vs SSIGP_ | .73 | .85 | .90 | N/A | N/A | N/A |
| *P*_SIGMA vs ST_ | .88 | .74 | .98 | N/A | N/A | N/A |
| *P* _SSIGP vs ST_ | .58 | .55 | .85 | N/A | N/A | N/A |
| 8-item Patient Health Questionnaire |  |  |  |  |  |  |
| SIGMA-Booster, estimated marginal means (SE) | 7.3 (0.6) | 6.5 (0.6) | 6.4 (0.7) | .11 | .19 | .98 |
| SIGMA, estimated marginal means (SE) | 6.3 (0.7) | 5.0 (0.7) | 5.5 (0.8) | .001 | .13 | .26 |
| SSIGP, estimated marginal means (SE) | 7.5 (0.8) | 6.1 (0.8) | 6.3 (0.8) | .02 | .03 | .76 |
| ST, estimated marginal means (SE) | 7.1 (0.6) | 5.9 (0.6) | 6.0 (0.6) | .002 | .02 | .67 |
| *P*_SIGMA-B vs SIGMA_ | .27 | .12 | .39 | *P*-value (interaction) | .97 | N/A |
| *P*_SIGMA-B vs SSIGP_ | .83 | .75 | .88 | N/A | N/A | N/A |
| *P*_SIGMA-B vs ST_ | .85 | .52 | .67 | N/A | N/A | N/A |
| *P*_SIGMA vs SSIGP_ | .24 | .30 | .51 | N/A | N/A | N/A |
| *P*_SIGMA vs ST_ | .38 | .36 | .63 | N/A | N/A | N/A |
| *P* _SSIGP vs ST_ | .69 | .80 | .81 | N/A | N/A | N/A |
| Suicidal/self-hurting thoughts |  |  |  |  |  |  |
| SIGMA-Booster, estimated marginal means (SE) | 0.3 (0.06) | 0.2 (0.05) | 0.3 (0.06) | .56 | .76 | .76 |
| SIGMA, estimated marginal means (SE) | 0.3 (0.06) | 0.2 (0.05) | 0.2 (0.06) | 0.06 | 0.051 | .74 |
| SSIGP, estimated marginal means (SE) | 0.3 (0.07) | 0.2 (0.06) | 0.2 (0.05) | .21 | .03 | .17 |
| ST, estimated marginal means (SE) | 0.3 (0.05) | 0.2 (0.05) | 0.2 (0.05) | .34 | .13 | .78 |
| *P*_SIGMA-B vs SIGMA_ | .87 | .43 | .47 | *P*-value (interaction) | .72 | N/A |
| *P*_SIGMA-B vs SSIGP_ | .62 | .99 | .35 | N/A | N/A | N/A |
| *P*_SIGMA-B vs ST_ | .86 | .61 | .37 | N/A | N/A | N/A |
| *P*_SIGMA vs SSIGP_ | .74 | .50 | .81 | N/A | N/A | N/A |
| *P*_SIGMA vs ST_ | .74 | .78 | .89 | N/A | N/A | N/A |
| *P* _SSIGP vs ST_ | .50 | .65 | .90 | N/A | N/A | N/A |
| Anxiety Control Questionnaire – Emotion Control |  |  |  |  |  |  |
| SIGMA-Booster, estimated marginal means (SE) | 13.4 (0.6) | 14.2 (0.5) | 13.4 (0.5) | .20 | .89 | .06 |
| SIGMA, estimated marginal means (SE) | 13.3 (0.7) | 14.9 (0.8) | 13.9 (0.9) | .008 | .30 | .06 |
| SSIGP, estimated marginal means (SE) | 13.9 (0.7) | 14.7 (0.6) | 14.4 (0.7) | .13 | .44 | .54 |
| ST, estimated marginal means (SE) | 13.8 (0.6) | 14.7 (0.6) | 15.1 (0.6) | .06 | .01 | .50 |
| *P*_SIGMA-B vs SIGMA_ | .89 | .41 | .56 | *P*-value (interaction) | .44 | N/A |
| *P*_SIGMA-B vs SSIGP_ | .60 | .51 | .24 | N/A | N/A | N/A |
| *P*_SIGMA-B vs ST_ | .67 | .53 | .02 | N/A | N/A | N/A |
| *P*_SIGMA vs SSIGP_ | .53 | .86 | .67 | N/A | N/A | N/A |
| *P*_SIGMA vs ST_ | .59 | .82 | .25 | N/A | N/A | N/A |
| *P* _SSIGP vs ST_ | .89 | .96 | .42 | N/A | N/A | N/A |
| Demoralisation Scale - Helplessness |  |  |  |  |  |  |
| SIGMA-Booster, estimated marginal means (SE) | 9.8 (0.4) | 9.6 (0.5) | 9.3 (0.5) | .58 | .27 | .52 |
| SIGMA, estimated marginal means (SE) | 9.6 (0.5) | 8.3 (0.5) | 8.8 (0.5) | .001 | .048 | .24 |
| SSIGP, estimated marginal means (SE) | 9.2 (0.6) | 8.8 (0.5) | 8.4 (0.5) | .38 | .07 | .35 |
| ST, estimated marginal means (SE) | 9.3 (0.4) | 8.2 (0.4) | 8.8 (0.5) | <.001 | .08 | .045 |
| *P*_SIGMA-B vs SIGMA_ | .77 | .050 | .42 | *P*-value (interaction) | .22 | N/A |
| *P*_SIGMA-B vs SSIGP_ | .43 | .27 | .20 | N/A | N/A | N/A |
| *P*_SIGMA-B vs ST_ | .42 | .03 | .41 | N/A | N/A | N/A |
| *P*_SIGMA vs SSIGP_ | .61 | .44 | .62 | N/A | N/A | N/A |
| *P*_SIGMA vs ST_ | .64 | .86 | .97 | N/A | N/A | N/A |
| *P* _SSIGP vs ST_ | .94 | .31 | .63 | N/A | N/A | N/A |
| Attitude towards Seeking Help |  |  |  |  |  |  |
| SIGMA-Booster, estimated marginal means (SE) | 19.0 (0.6) | 20.2 (0.6) | 20.8 (0.6) | .02 | .001 | .20 |
| SIGMA, estimated marginal means (SE) | 19.5 (0.9) | 21.3 (1.0) | 21.0 (1.0) | .004 | .01 | .59 |
| SSIGP, estimated marginal means (SE) | 18.9 (0.8) | 21.5 (0.7) | 20.5 (0.8) | <.001 | .02 | .09 |
| ST, estimated marginal means (SE) | 19.8 (0.7) | 21.9 (0.7) | 21.0 (0.8) | .001 | .047 | .11 |
| *P*_SIGMA-B vs SIGMA_ | .61 | .30 | .81 | *P*-value (interaction) | .29 | N/A |
| *P*_SIGMA-B vs SSIGP_ | .86 | .16 | .78 | N/A | N/A | N/A |
| *P*_SIGMA-B vs ST_ | .39 | .054 | .79 | N/A | N/A | N/A |
| *P*_SIGMA vs SSIGP_ | .56 | .88 | .67 | N/A | N/A | N/A |
| *P*_SIGMA vs ST_ | .85 | .59 | .99 | N/A | N/A | N/A |
| *P* _SSIGP vs ST_ | .38 | .66 | .64 | N/A | N/A | N/A |
| Warwick-Edinburgh Mental Well-being Scale |  |  |  |  |  |  |
| SIGMA-Booster, estimated marginal means (SE) | 41.6 (1.2) | 43.2 (1.1) | 45.6 (1.1) | .08 | <.001 | .004 |
| SIGMA, estimated marginal means (SE) | 42.7 (1.5) | 44.0 (1.7) | 45.3 (1.7) | .09 | .07 | .31 |
| SSIGP, estimated marginal means (SE) | 43.8 (1.6) | 45.8 (1.6) | 45.9 (1.4) | .11 | .06 | .91 |
| ST, estimated marginal means (SE) | 44.6 (1.3) | 47.2 (1.4) | 48.6 (1.3) | .002 | <.001 | .11 |
| *P*_SIGMA-B vs SIGMA_ | .55 | .66 | .90 | *P*-value (interaction) | .64 | N/A |
| *P*_SIGMA-B vs SSIGP_ | .24 | .17 | .85 | N/A | N/A | N/A |
| *P*_SIGMA-B vs ST_ | .08 | .02 | .07 | N/A | N/A | N/A |
| *P*_SIGMA vs SSIGP_ | .59 | .44 | .79 | N/A | N/A | N/A |
| *P*_SIGMA vs ST_ | .33 | .15 | .12 | N/A | N/A | N/A |
| *P* _SSIGP vs ST_ | .69 | .49 | .15 | N/A | N/A | N/A |

^a^SIGMA-Booster: SIGMA with boosters.

^b^SIGMA: single-session intervention of growth mindset for anxiety.

^c^SSIGP: single-session intervention of growth mindset of personality.

^d^ST: support therapy.
