# Supplementary material for: Effects of Web-Based Single-Session Growth Mindset Interventions for Reducing Adolescent Anxiety: Four-Armed Randomized Controlled Trial
Source: JMIR Pediatr Parent. 2025 Apr 18;8:e63500. doi: 10.2196/63500 (PMC12048788; doi:10.2196/63500)
Supplement: Multimedia Appendix 4 [file pediatrics_v8i1e63500_app4.docx]

**Multimedia Appendix 4.** Moderation effects of baseline anxiety levels in the treatment effects

| **7-item Generalized Anxiety Disorder** | Baseline | 2-week follow-up | 8-week follow-up | *P*-value (Baseline  vs 2-week follow-up) | *P*-value (Baseline vs 8-week follow-up) | *P*-value  (2-week  vs 8-week follow-up) |
| --- | --- | --- | --- | --- | --- | --- |
| High anxiety level |  |  |  |  |  |  |
| SIGMA-Booster^a^, estimated marginal means (SE) | 13.8 (0.6) | 10.8 (0.9) | 9.6 (0.9) | .002 | <.001 | .19 |
| SIGMA^b^, estimated marginal means (SE) | 13.8 (0.5) | 10.8 (0.8) | 8.8 (0.8) | <.001 | <.001 | .004 |
| SSIGP^c^, estimated marginal means (SE) | 13.5 (0.5) | 9.5 (0.8) | 9.9 (0.9) | <.001 | <.001 | .60 |
| ST^d^, estimated marginal means (SE) | 14.8 (0.5) | 11.7 (0.9) | 10.8 (0.8) | <.001 | <.001 | .29 |
| *P*_SIGMA-B vs SIGMA_ | 1.00 | .97 | .51 | *P*-value  (interaction) | .49 | N/A |
| *P*_SIGMA-B vs SSIGP_ | .66 | .28 | .83 | N/A | N/A | N/A |
| *P*_SIGMA-B vs ST_ | .18 | .44 | .34 | N/A | N/A | N/A |
| *P*_SIGMA vs SSIGP_ | .62 | .23 | .37 | N/A | N/A | N/A |
| *P*_SIGMA vs ST_ | .13 | .44 | .09 | N/A | N/A | N/A |
| *P* _SSIGP vs ST_ | .050 | .054 | .45 | N/A | N/A | N/A |
| Low anxiety level |  |  |  |  |  |  |
| SIGMA-Booster, estimated marginal means (SE) | 4.5 (0.3) | 4.4 (0.4) | 4.7 (0.4) | .59 | .65 | .37 |
| SIGMA, estimated marginal means (SE) | 4.0 (0.2) | 3.9 (0.3) | 4.0 (0.4) | .83 | .86 | .72 |
| SSIGP, estimated marginal means (SE) | 4.6 (0.3) | 3.9 (0.4) | 4.1 (0.4) | .02 | .14 | .53 |
| ST, estimated marginal means (SE) | 4.0 (0.2) | 3.4 (0.3) | 4.1 (0.3) | .04 | .85 | .048 |
| *P*_SIGMA-B vs SIGMA_ | .09 | .37 | .21 | *P*-value  (interaction) | .56 | N/A |
| *P*_SIGMA-B vs SSIGP_ | .97 | .34 | .25 | N/A | N/A | N/A |
| *P*_SIGMA-B vs ST_ | .12 | .046 | .22 | N/A | N/A | N/A |
| *P*_SIGMA vs SSIGP_ | .10 | .93 | .90 | N/A | N/A | N/A |
| *P*_SIGMA vs ST_ | .94 | .30 | .95 | N/A | N/A | N/A |
| *P* _SSIGP vs ST_ | .11 | .37 | .95 | N/A | N/A | N/A |
| **8-item Patient Health Questionnaire** |  |  |  |  |  |  |
| High anxiety level |  |  |  |  |  |  |
| SIGMA-Booster, estimated marginal means (SE) | 13.4 (0.8) | 11.9 (0.9) | 10.2 (0.9) | .10 | < .001 | .02 |
| SIGMA, estimated marginal means (SE) | 13.2 (0.7) | 10.6 (0.8) | 9.3 (0.9) | .005 | < .001 | .07 |
| SSIGP, estimated marginal means (SE) | 12.7 (0.8) | 10.1 (1.0) | 10.1 (0.9) | .005 | .001 | .99 |
| ST, estimated marginal means (SE) | 14.0 (0.7) | 12.9 (0.9) | 11.5 (0.9) | .18 | .008 | .08 |
| *P*_SIGMA-B vs SIGMA_ | .83 | .31 | .52 | *P*-value  (interaction) | .60 | N/A |
| *P*_SIGMA-B vs SSIGP_ | .53 | .19 | .98 | N/A | N/A | N/A |
| *P*_SIGMA-B vs ST_ | .60 | .42 | .31 | N/A | N/A | N/A |
| *P*_SIGMA vs SSIGP_ | .65 | .68 | .52 | N/A | N/A | N/A |
| *P*_SIGMA vs ST_ | .40 | .06 | .09 | N/A | N/A | N/A |
| *P* _SSIGP vs ST_ | .21 | .03 | .28 | N/A | N/A | N/A |
| Low anxiety level |  |  |  |  |  |  |
| SIGMA-Booster, estimated marginal means (SE) | 5.4 (0.4) | 4.6 (0.4) | 4.5 (0.4) | .06 | .03 | .80 |
| SIGMA, estimated marginal means (SE) | 5.1 (0.3) | 4.2 (0.4) | 4.3 (0.4) | .004 | .04 | .71 |
| SSIGP, estimated marginal means (SE) | 5.2 (0.4) | 4.0 (0.4) | 4.3 (0.4) | .002 | .01 | .39 |
| ST, estimated marginal means (SE) | 5.1(0.3) | 3.7 (0.3) | 4.2 (0.4) | < .001 | .04 | .11 |
| *P*_SIGMA-B vs SIGMA_ | .52 | .46 | .78 | *P*-value  (interaction) | .85 | N/A |
| *P*_SIGMA-B vs SSIGP_ | .76 | .34 | .84 | N/A | N/A | N/A |
| *P*_SIGMA-B vs ST_ | .49 | .06 | .71 | N/A | N/A | N/A |
| *P*_SIGMA vs SSIGP_ | .75 | .80 | .95 | N/A | N/A | N/A |
| *P*_SIGMA vs ST_ | .96 | .28 | .91 | N/A | N/A | N/A |
| *P* _SSIGP vs ST_ | .71 | .46 | .86 | N/A | N/A | N/A |
| **Suicidal/self-hurting thoughts** |  |  |  |  |  |  |
| High anxiety level |  |  |  |  |  |  |
| SIGMA-Booster, estimated marginal means (SE) | 0.7 (0.1) | 0.6 (0.1) | 0.5 (0.1) | .24 | .004 | .30 |
| SIGMA, estimated marginal means (SE) | 0.7 (0.1) | 0.6 (0.1) | 0.5 (0.1) | .06 | .01 | .48 |
| SSIGP, estimated marginal means (SE) | 0.6 (0.1) | 0.5 (0.1) | 0.4 (0.1) | .91 | .004 | .03 |
| ST, estimated marginal means (SE) | 0.7 (0.1) | 0.6 (0.1) | 0.6 (0.1) | .76 | .38 | .46 |
| *P*_SIGMA-B vs SIGMA_ | .89 | .63 | .83 | *P*-value  (interaction) | .78 | N/A |
| *P*_SIGMA-B vs SSIGP_ | .13 | .48 | .09 | N/A | N/A | N/A |
| *P*_SIGMA-B vs ST_ | .63 | .87 | .66 | N/A | N/A | N/A |
| *P*_SIGMA vs SSIGP_ | .15 | .79 | .11 | N/A | N/A | N/A |
| *P*_SIGMA vs ST_ | .71 | .49 | .48 | N/A | N/A | N/A |
| *P* _SSIGP vs ST_ | .29 | .37 | .02 | N/A | N/A | N/A |
| Low anxiety level |  |  |  |  |  |  |
| SIGMA-Booster, estimated marginal means (SE) | 0.2 (0.04) | 0.2 (0.04) | 0.2 (0.04) | .12 | .15 | .93 |
| SIGMA, estimated marginal means (SE) | 0.3 (0.04) | 0.2 (0.04) | 0.2 (0.03) | .19 | .23 | .89 |
| SSIGP, estimated marginal means (SE) | 0.2 (0.04) | 0.2 (0.04) | 0.2 (0.04) | .049 | .02 | .69 |
| ST, estimated marginal means (SE) | 0.2 (0.03) | 0.2 (0.03) | 0.2 (0.03) | .52 | .87 | .60 |
| *P*_SIGMA-B vs SIGMA_ | .12 | .47 | .58 | *P*-value  (interaction) | .10 | N/A |
| *P*_SIGMA-B vs SSIGP_ | .26 | .13 | .09 | N/A | N/A | N/A |
| *P*_SIGMA-B vs ST_ | .49 | .18 | .10 | N/A | N/A | N/A |
| *P*_SIGMA vs SSIGP_ | .77 | .38 | .20 | N/A | N/A | N/A |
| *P*_SIGMA vs ST_ | .02 | .51 | .23 | N/A | N/A | N/A |
| *P* _SSIGP vs ST_ | .07 | .83 | .92 | N/A | N/A | N/A |
| **Anxiety Control Questionnaire – Emotion Control** |  |  |  |  |  |  |
| High anxiety level |  |  |  |  |  |  |
| SIGMA-Booster, estimated marginal means (SE) | 10.0 (0.7) | 13.2 (0.6) | 11.6 (0.6) | < .001 | .01 | .02 |
| SIGMA, estimated marginal means (SE) | 9.9 (0.6) | 11.4 (0.6) | 11.1 (0.7) | .06 | .21 | .68 |
| SSIGP, estimated marginal means (SE) | 10.3 (0.7) | 10.7 (0.7) | 10.6 (0.6) | .48 | .69 | .89 |
| ST, estimated marginal means (SE) | 9.9 (0.7) | 12.6 (0.7) | 12.0 (0.6) | < .001 | .004 | .39 |
| *P*_SIGMA-B vs SIGMA_ | .99 | .04 | .59 | *P*-value  (interaction) | .06 | N/A |
| *P*_SIGMA-B vs SSIGP_ | .72 | .007 | .27 | N/A | N/A | N/A |
| *P*_SIGMA-B vs ST_ | .93 | .56 | .61 | N/A | N/A | N/A |
| *P*_SIGMA vs SSIGP_ | .69 | .46 | .64 | N/A | N/A | N/A |
| *P*_SIGMA vs ST_ | .93 | .16 | .31 | N/A | N/A | N/A |
| *P* _SSIGP vs ST_ | .66 | .04 | .11 | N/A | N/A | N/A |
| Low anxiety level |  |  |  |  |  |  |
| SIGMA-Booster, estimated marginal means (SE) | 15.0 (0.4) | 14.3 (0.4) | 14.2 (0.4) | .10 | .11 | .98 |
| SIGMA, estimated marginal means (SE) | 15.0 (0.4) | 14.8 (0.4) | 14.4 (0.4) | .76 | .22 | .37 |
| SSIGP, estimated marginal means (SE) | 15.1 (0.4) | 15.5 (0.4) | 14.4 (0.5) | .27 | .17 | .02 |
| ST, estimated marginal means (SE) | 14.4 (0.4) | 14.7 (0.4) | 15.2 (0.5) | .56 | .14 | .39 |
| *P*_SIGMA-B vs SIGMA_ | .97 | .31 | .73 | *P*-value  (interaction) | .10 | N/A |
| *P*_SIGMA-B vs SSIGP_ | .89 | .03 | .80 | N/A | N/A | N/A |
| *P*_SIGMA-B vs ST_ | .33 | .45 | .13 | N/A | N/A | N/A |
| *P*_SIGMA vs SSIGP_ | .85 | .27 | .95 | N/A | N/A | N/A |
| *P*_SIGMA vs ST_ | .31 | .82 | .25 | N/A | N/A | N/A |
| *P* _SSIGP vs ST_ | .26 | .19 | .26 | N/A | N/A | N/A |
| **Demoralisation Scale - Helplessness** |  |  |  |  |  |  |
| High anxiety level |  |  |  |  |  |  |
| SIGMA-Booster, estimated marginal means (SE) | 13.3 (0.4) | 12.3 (0.5) | 11.9 (0.6) | .04 | .004 | .36 |
| SIGMA, estimated marginal means (SE) | 13.0 (0.4) | 11.2 (0.6) | 11.0 (0.6) | .006 | .001 | .69 |
| SSIGP, estimated marginal means (SE) | 12.3 (0.6) | 11.1 (0.5) | 11.5 (0.5) | .054 | .22 | .54 |
| ST, estimated marginal means (SE) | 13.8 (0.5) | 12.5 (0.6) | 12.9 (0.6) | .02 | .08 | .41 |
| *P*_SIGMA-B vs SIGMA_ | .65 | .17 | .26 | *P*-value  (interaction) | .67 | N/A |
| *P*_SIGMA-B vs SSIGP_ | .17 | .11 | .58 | N/A | N/A | N/A |
| *P*_SIGMA-B vs ST_ | .47 | .76 | .20 | N/A | N/A | N/A |
| *P*_SIGMA vs SSIGP_ | .30 | .87 | .56 | N/A | N/A | N/A |
| *P*_SIGMA vs ST_ | .24 | .11 | .02 | N/A | N/A | N/A |
| *P* _SSIGP vs ST_ | .045 | .07 | .07 | N/A | N/A | N/A |
| Low anxiety level |  |  |  |  |  |  |
| SIGMA-Booster, estimated marginal means (SE) | 8.6 (0.3) | 8.7 (0.3) | 8.5 (0.3) | .72 | .82 | .54 |
| SIGMA, estimated marginal means (SE) | 9.1 (0.3) | 8.2 (0.3) | 8.4 (0.3) | .006 | .06 | .57 |
| SSIGP, estimated marginal means (SE) | 8.5 (0.3) | 8.4 (0.4) | 8.0 (0.3) | .62 | .04 | .26 |
| ST, estimated marginal means (SE) | 8.4 (0.3) | 7.6 (0.3) | 7.8 (0.3) | .004 | .03 | .60 |
| *P*_SIGMA-B vs SIGMA_ | .20 | .29 | .81 | *P*-value  (interaction) | .23 | N/A |
| *P*_SIGMA-B vs SSIGP_ | .80 | .44 | .20 | N/A | N/A | N/A |
| *P*_SIGMA-B vs ST_ | .65 | .01 | .08 | N/A | N/A | N/A |
| *P*_SIGMA vs SSIGP_ | .16 | .82 | .30 | N/A | N/A | N/A |
| *P*_SIGMA vs ST_ | .09 | .16 | .14 | N/A | N/A | N/A |
| *P* _SSIGP vs ST_ | .87 | .11 | .64 | N/A | N/A | N/A |
| **Attitude towards Seeking Help** |  |  |  |  |  |  |
| High anxiety level |  |  |  |  |  |  |
| SIGMA-Booster, estimated marginal means (SE) | 18.1 (0.7) | 19.2 (0.8) | 19.2 (0.7) | .09 | .07 | .98 |
| SIGMA, estimated marginal means (SE) | 17.2 (0.8) | 18.7 (0.7) | 19.0 (0.6) | .046 | .02 | .71 |
| SSIGP, estimated marginal means (SE) | 18.6 (0.8) | 19.9 (0.7) | 18.6 (0.6) | .09 | .95 | .04 |
| ST, estimated marginal means (SE) | 17.0 (0.7) | 20.1 (0.6) | 19.5 (0.8) | < .001 | .005 | .33 |
| *P*_SIGMA-B vs SIGMA_ | .38 | .66 | .79 | *P*-value  (interaction) | .12 | N/A |
| *P*_SIGMA-B vs SSIGP_ | .60 | .48 | .50 | N/A | N/A | N/A |
| *P*_SIGMA-B vs ST_ | .26 | .38 | .82 | N/A | N/A | N/A |
| *P*_SIGMA vs SSIGP_ | .19 | .19 | .64 | N/A | N/A | N/A |
| *P*_SIGMA vs ST_ | .85 | .13 | .61 | N/A | N/A | N/A |
| *P* _SSIGP vs ST_ | .11 | .82 | .36 | N/A | N/A | N/A |
| Low anxiety level |  |  |  |  |  |  |
| SIGMA-Booster, estimated marginal means (SE) | 19.6 (0.4) | 20.3 (0.4) | 20.4 (0.4) | .06 | .049 | .68 |
| SIGMA, estimated marginal means (SE) | 20.1 (0.5) | 20.8 (0.4) | 20.4 (0.4) | .051 | .52 | .10 |
| SSIGP, estimated marginal means (SE) | 18.8 (0.5) | 20.5 (0.5) | 20.4 (0.5) | < .001 | .001 | .72 |
| ST, estimated marginal means (SE) | 20.3 (0.5) | 21.1 (0.5) | 20.1 (0.5) | .07 | .60 | .02 |
| *P*_SIGMA-B vs SIGMA_ | .39 | .32 | .91 | *P*-value  (interaction) | .07 | N/A |
| *P*_SIGMA-B vs SSIGP_ | .20 | .71 | .92 | N/A | N/A | N/A |
| *P*_SIGMA-B vs ST_ | .25 | .18 | .59 | N/A | N/A | N/A |
| *P*_SIGMA vs SSIGP_ | .04 | .58 | 1.0 | N/A | N/A | N/A |
| *P*_SIGMA vs ST_ | .77 | .68 | .67 | N/A | N/A | N/A |
| *P* _SSIGP vs ST_ | .02 | .37 | .69 | N/A | N/A | N/A |
| **Warwick-Edinburgh Mental Well-being Scale** |  |  |  |  |  |  |
| High anxiety level |  |  |  |  |  |  |
| SIGMA-Booster, estimated marginal means (SE) | 34.2 (1.1) | 37.6 (1.7) | 40.4 (1.4) | .03 | < .001 | .08 |
| SIGMA, estimated marginal means (SE) | 36.0 (1.1) | 35.5 (1.5) | 38.9 (1.8) | .72 | .12 | .03 |
| SSIGP, estimated marginal means (SE) | 35.9 (1.3) | 38.4 (1.7) | 39.0 (1.4) | .15 | .02 | .67 |
| ST, estimated marginal means (SE) | 35.5 (1.3) | 38.5 (1.5) | 39.5 (1.5) | .09 | .04 | .51 |
| *P*_SIGMA-B vs SIGMA_ | .25 | .37 | .50 | *P*-value  (interaction) | .24 | N/A |
| *P*_SIGMA-B vs SSIGP_ | .32 | .73 | .49 | N/A | N/A | N/A |
| *P*_SIGMA-B vs ST_ | .47 | .69 | .66 | N/A | N/A | N/A |
| *P*_SIGMA vs SSIGP_ | .94 | .20 | .94 | N/A | N/A | N/A |
| *P*_SIGMA vs ST_ | .75 | .16 | .79 | N/A | N/A | N/A |
| *P* _SSIGP vs ST_ | .81 | .97 | .83 | N/A | N/A | N/A |
| Low anxiety level |  |  |  |  |  |  |
| SIGMA-Booster, estimated marginal means (SE) | 44.6 (0.9) | 45.0 (0.9) | 45.4 (1.0) | .56 | .33 | .56 |
| SIGMA, estimated marginal means (SE) | 45.0 (0.9) | 46.4 (1.0) | 46.7 (1.1) | .07 | .08 | .73 |
| SSIGP, estimated marginal means (SE) | 45.8 (1.0) | 46.2 (1.1) | 46.3 (1.1) | .73 | .65 | .91 |
| ST, estimated marginal means (SE) | 45.7 (0.9) | 47.3 (1.0) | 48.9 (1.0) | .06 | < .001 | .03 |
| *P*_SIGMA-B vs SIGMA_ | .68 | .25 | .34 | *P*-value  (interaction) | .35 | N/A |
| *P*_SIGMA-B vs SSIGP_ | .31 | .39 | .53 | N/A | N/A | N/A |
| *P*_SIGMA-B vs ST_ | .35 | .09 | .01 | N/A | N/A | N/A |
| *P*_SIGMA vs SSIGP_ | .55 | .85 | .77 | N/A | N/A | N/A |
| *P*_SIGMA vs ST_ | .60 | .56 | .15 | N/A | N/A | N/A |
| *P* _SSIGP vs ST_ | .92 | .45 | .08 | N/A | N/A | N/A |

^a^SIGMA-Booster: SIGMA with boosters.

^b^SIGMA: single-session intervention of growth mindset for anxiety.

^c^SSIGP: single-session intervention of growth mindset of personality.

^d^ST: support therapy.

.
