# Supplementary material for: Effects of Web-Based Single-Session Growth Mindset Interventions for Reducing Adolescent Anxiety: Four-Armed Randomized Controlled Trial
Source: JMIR Pediatr Parent. 2025 Apr 18;8:e63500. doi: 10.2196/63500 (PMC12048788; doi:10.2196/63500)
Supplement: Multimedia Appendix 5 [file pediatrics_v8i1e63500_app5.docx]

**Multimedia Appendix 5**. Moderation effects of baseline motivation levels in the treatment effects

| **7-item Generalized Anxiety Disorder** | Baseline | 2-week follow-up | 8-week follow-up | *P*-value  (Baseline  vs 2-week follow-up) | *P*-value  (Baseline vs 8-week follow-up) | *P*-value  (2-week  vs 8-week follow-up) |
| --- | --- | --- | --- | --- | --- | --- |
| High motivation level |  |  |  |  |  |  |
| SIGMA-Booster^a^, estimated marginal means (SE) | 7.7 (0.5) | 6.2 (0.6) | 6.6 (0.6) | .004 | .04 | .54 |
| SIGMA^b^, estimated marginal means (SE) | 6.7 (0.5) | 5.8 (0.6) | 5.4 (0.5) | .01 | .004 | .38 |
| SSIGP^c^, estimated marginal means (SE) | 7.2 (0.5) | 5.6 (0.5) | 6.0 (0.5) | <.001 | .007 | .31 |
| ST^d^, estimated marginal means (SE) | 7.7 (0.6) | 6.6 (0.6) | 6.6 (0.5) | .002 | .007 | .96 |
| *P*_SIGMA-B vs SIGMA_ | .20 | .56 | .13 | *P*-value  (interaction) | .86 | N/A |
| *P*_SIGMA-B vs SSIGP_ | .50 | .39 | .49 | N/A | N/A | N/A |
| *P*_SIGMA-B vs ST_ | .97 | .68 | .97 | N/A | N/A | N/A |
| *P*_SIGMA vs SSIGP_ | .55 | .79 | .39 | N/A | N/A | N/A |
| *P*_SIGMA vs ST_ | .21 | .32 | .11 | N/A | N/A | N/A |
| *P* _SSIGP vs ST_ | .50 | .20 | .45 | N/A | N/A | N/A |
| Low motivation level |  |  |  |  |  |  |
| SIGMA-Booster, estimated marginal means (SE) | 5.8 (0.6) | 5.6 (0.6) | 5.2 (0.6) | .73 | .28 | .40 |
| SIGMA, estimated marginal means (SE) | 6.4 (0.6) | 5.8 (0.6) | 5.3 (0.5) | .16 | .04 | .24 |
| SSIGP, estimated marginal means (SE) | 6.7 (0.6) | 5.2 (0.6) | 5.2 (0.6) | .002 | .005 | 1.0 |
| ST, estimated marginal means (SE) | 6.0 (0.5) | 4.6 (0.5) | 5.0 (0.6) | .003 | .11 | .51 |
| *P*_SIGMA-B vs SIGMA_ | .46 | .87 | .84 | *P*-value  (interaction) | .48 | N/A |
| *P*_SIGMA-B vs SSIGP_ | .26 | .61 | .97 | N/A | N/A | N/A |
| *P*_SIGMA-B vs ST_ | .79 | .23 | .87 | N/A | N/A | N/A |
| *P*_SIGMA vs SSIGP_ | .67 | .50 | .87 | N/A | N/A | N/A |
| *P*_SIGMA vs ST_ | .65 | .17 | .71 | N/A | N/A | N/A |
| *P* _SSIGP vs ST_ | .39 | .51 | .84 | N/A | N/A | N/A |
| **8-item Patient Health Questionnaire** |  |  |  |  |  |  |
| High motivation level |  |  |  |  |  |  |
| SIGMA-Booster, estimated marginal means (SE) | 7.6 (0.6) | 6.8 (0.6) | 6.3 (0.6) | .09 | .02 | .25 |
| SIGMA, estimated marginal means (SE) | 7.3 (0.5) | 6.1 (0.6) | 5.6 (0.6) | .004 | .002 | .32 |
| SSIGP, estimated marginal means (SE) | 7.3 (0.5) | 5.7 (0.6) | 6.6 (0.6) | .001 | .10 | .04 |
| ST, estimated marginal means (SE) | 8.3 (0.6) | 7.1 (0.6) | 6.7 (0.6) | .004 | <.001 | .33 |
| *P*_SIGMA-B vs SIGMA_ | .72 | .39 | .36 | *P*-value  (interaction) | .26 | N/A |
| *P*_SIGMA-B vs SSIGP_ | .73 | .21 | .78 | N/A | N/A | N/A |
| *P*_SIGMA-B vs ST_ | .37 | .70 | .68 | N/A | N/A | N/A |
| *P*_SIGMA vs SSIGP_ | .99 | .69 | .23 | N/A | N/A | N/A |
| *P*_SIGMA vs ST_ | .18 | .23 | .17 | N/A | N/A | N/A |
| *P* _SSIGP vs ST_ | .18 | .11 | .89 | N/A | N/A | N/A |
| Low motivation level |  |  |  |  |  |  |
| SIGMA-Booster, estimated marginal means (SE) | 7.3 (0.6) | 6.0 (0.7) | 5.4 (0.6) | .07 | .003 | .33 |
| SIGMA, estimated marginal means (SE) | 7.2 (0.6) | 5.7 (0.6) | 5.8 (0.6) | .005 | .01 | .82 |
| SSIGP, estimated marginal means (SE) | 7.2 (0.7) | 5.7 (0.7) | 5.1 (0.6) | .01 | <.001 | .29 |
| ST, estimated marginal means (SE) | 6.4 (0.5) | 5.0 (0.6) | 5.6 (0.6) | .004 | .20 | .36 |
| *P*_SIGMA-B vs SIGMA_ | .93 | .74 | .63 | *P*-value  (interaction) | .69 | N/A |
| *P*_SIGMA-B vs SSIGP_ | .99 | .74 | .69 | N/A | N/A | N/A |
| *P*_SIGMA-B vs ST_ | .31 | .27 | .86 | N/A | N/A | N/A |
| *P*_SIGMA vs SSIGP_ | .94 | .97 | .34 | N/A | N/A | N/A |
| *P*_SIGMA vs ST_ | .36 | .41 | .79 | N/A | N/A | N/A |
| *P* _SSIGP vs ST_ | .35 | .47 | .56 | N/A | N/A | N/A |
| **Suicidal/self-hurting thoughts** |  |  |  |  |  |  |
| High motivation level |  |  |  |  |  |  |
| SIGMA-Booster, estimated marginal means (SE) | 0.3 (0.05) | 0.4 (0.05) | 0.3 (0.05) | .11 | .40 | .45 |
| SIGMA, estimated marginal means (SE) | 0.4 (0.05) | 0.3 (0.05) | 0.3 (0.04) | .07 | .01 | .44 |
| SSIGP, estimated marginal means (SE) | 0.3 (0.05) | 0.3 (0.05) | 0.2 (0.05) | .36 | .07 | .44 |
| ST, estimated marginal means (SE) | 0.3 (0.04) | 0.3 (0.04) | 0.3 (0.05) | .70 | .66 | .36 |
| *P*_SIGMA-B vs SIGMA_ | .13 | .44 | .47 | *P*-value  (interaction) | .07 | N/A |
| *P*_SIGMA-B vs SSIGP_ | .61 | .21 | .21 | N/A | N/A | N/A |
| *P*_SIGMA-B vs ST_ | .98 | .18 | .80 | N/A | N/A | N/A |
| *P*_SIGMA vs SSIGP_ | .39 | .58 | .57 | N/A | N/A | N/A |
| *P*_SIGMA vs ST_ | .14 | .52 | .62 | N/A | N/A | N/A |
| *P* _SSIGP vs ST_ | .62 | .96 | .31 | N/A | N/A | N/A |
| Low motivation level |  |  |  |  |  |  |
| SIGMA-Booster, estimated marginal means (SE) | 0.4 (0.06) | 0.3 (0.06) | 0.3 (0.06) | .38 | .39 | 1.0 |
| SIGMA, estimated marginal means (SE) | 0.4 (0.06) | 0.3 (0.05) | 0.3 (0.05) | .24 | .36 | .67 |
| SSIGP, estimated marginal means (SE) | 0.3 (0.06) | 0.3 (0.06) | 0.2 (0.05) | .26 | .001 | .07 |
| ST, estimated marginal means (SE) | 0.3 (0.05) | 0.3 (0.05) | 0.2 (0.05) | .36 | .20 | .04 |
| *P*_SIGMA-B vs SIGMA_ | .85 | .87 | .91 | *P*-value  (interaction) | .16 | N/A |
| *P*_SIGMA-B vs SSIGP_ | .76 | .50 | .04 | N/A | N/A | N/A |
| *P*_SIGMA-B vs ST_ | .35 | .75 | .24 | N/A | N/A | N/A |
| *P*_SIGMA vs SSIGP_ | .61 | .58 | .02 | N/A | N/A | N/A |
| *P*_SIGMA vs ST_ | .25 | .63 | .18 | N/A | N/A | N/A |
| *P* _SSIGP vs ST_ | .56 | .32 | .33 | N/A | N/A | N/A |
| **Anxiety Control Questionnaire – Emotion Control** |  |  |  |  |  |  |
| High motivation level |  |  |  |  |  |  |
| SIGMA-Booster, estimated marginal means (SE) | 13.0 (0.5) | 13.3 (0.4) | 12.9 (0.4) | .55 | .90 | .40 |
| SIGMA, estimated marginal means (SE) | 13.1 (0.4) | 14.3 (0.5) | 13.5 (0.6) | .02 | .45 | .17 |
| SSIGP, estimated marginal means (SE) | 13.1 (0.5) | 13.8 (0.5) | 13.6 (0.5) | .09 | .30 | .76 |
| ST, estimated marginal means (SE) | 13.1 (0.5) | 14.3 (0.5) | 14.3 (0.5) | .005 | .02 | .91 |
| *P*_SIGMA-B vs SIGMA_ | .86 | .14 | .39 | *P*-value  (interaction) | .60 | N/A |
| *P*_SIGMA-B vs SSIGP_ | .88 | .51 | .32 | N/A | N/A | N/A |
| *P*_SIGMA-B vs ST_ | .92 | .13 | .04 | N/A | N/A | N/A |
| *P*_SIGMA vs SSIGP_ | .99 | .49 | .92 | N/A | N/A | N/A |
| *P*_SIGMA vs ST_ | .94 | .97 | .33 | N/A | N/A | N/A |
| *P* _SSIGP vs ST_ | .96 | .45 | .37 | N/A | N/A | N/A |
| Low motivation level |  |  |  |  |  |  |
| SIGMA-Booster, estimated marginal means (SE) | 14.7 (0.6) | 14.9 (0.6) | 14.4 (0.6) | .77 | .65 | .35 |
| SIGMA, estimated marginal means (SE) | 14.3 (0.5) | 13.4 (0.6) | 13.5 (0.5) | .12 | .22 | .83 |
| SSIGP, estimated marginal means (SE) | 14.6 (0.6) | 14.8 (0.6) | 13.0 (0.7) | .71 | .02 | .008 |
| ST, estimated marginal means (SE) | 13.3 (0.6) | 13.9 (0.6) | 14.4 (0.6) | .41 | .13 | .53 |
| *P*_SIGMA-B vs SIGMA_ | .59 | .06 | .28 | *P*-value  (interaction) | .08 | N/A |
| *P*_SIGMA-B vs SSIGP_ | .88 | .90 | .11 | N/A | N/A | N/A |
| *P*_SIGMA-B vs ST_ | .12 | .23 | .98 | N/A | N/A | N/A |
| *P*_SIGMA vs SSIGP_ | .68 | .08 | .50 | N/A | N/A | N/A |
| *P*_SIGMA vs ST_ | .24 | .52 | .29 | N/A | N/A | N/A |
| *P* _SSIGP vs ST_ | .14 | .29 | .13 | N/A | N/A | N/A |
| **Demoralisation Scale - Helplessness** |  |  |  |  |  |  |
| High motivation level |  |  |  |  |  |  |
| SIGMA-Booster, estimated marginal means (SE) | 10.0 (0.4) | 9.7 (0.4) | 9.5 (0.4) | .36 | .10 | .57 |
| SIGMA, estimated marginal means (SE) | 10.2 (0.4) | 9.0 (0.4) | 8.6 (0.4) | .002 | <.001 | .44 |
| SSIGP, estimated marginal means (SE) | 9.7 (0.4) | 9.1 (0.4) | 9.1 (0.4) | .09 | .07 | .98 |
| ST, estimated marginal means (SE) | 10.5 (0.4) | 9.4 (0.4) | 10.0 (0.4) | .001 | .09 | .10 |
| *P*_SIGMA-B vs SIGMA_ | .75 | .19 | .10 | *P*-value  (interaction) | .24 | N/A |
| *P*_SIGMA-B vs SSIGP_ | .60 | .30 | .46 | N/A | N/A | N/A |
| *P*_SIGMA-B vs ST_ | .36 | .64 | .44 | N/A | N/A | N/A |
| *P*_SIGMA vs SSIGP_ | .40 | .79 | .38 | N/A | N/A | N/A |
| *P*_SIGMA vs ST_ | .53 | .43 | .03 | N/A | N/A | N/A |
| *P* _SSIGP vs ST_ | .16 | .59 | .15 | N/A | N/A | N/A |
| Low motivation level |  |  |  |  |  |  |
| SIGMA-Booster, estimated marginal means (SE) | 9.5 (0.4) | 9.5 (0.5) | 9.2 (0.5) | .90 | .58 | .46 |
| SIGMA, estimated marginal means (SE) | 10.1 (0.4) | 9.1 (0.4) | 9.7 (0.5) | .02 | .34 | .17 |
| SSIGP, estimated marginal means (SE) | 9.3 (0.5) | 9.1 (0.5) | 8.6 (0.5) | .71 | .13 | .30 |
| ST, estimated marginal means (SE) | 9.1 (0.4) | 8.4 (0.4) | 8.2 (0.4) | .04 | .03 | .69 |
| *P*_SIGMA-B vs SIGMA_ | .23 | .54 | .45 | *P*-value  (interaction) | .43 | N/A |
| *P*_SIGMA-B vs SSIGP_ | .75 | .54 | .40 | N/A | N/A | N/A |
| *P*_SIGMA-B vs ST_ | .55 | .08 | .14 | N/A | N/A | N/A |
| *P*_SIGMA vs SSIGP_ | .17 | .96 | .09 | N/A | N/A | N/A |
| *P*_SIGMA vs ST_ | .09 | .22 | .02 | N/A | N/A | N/A |
| *P* _SSIGP vs ST_ | .82 | .27 | .50 | N/A | N/A | N/A |
| **Attitude towards Seeking Help** |  |  |  |  |  |  |
| High motivation level |  |  |  |  |  |  |
| SIGMA-Booster, estimated marginal means (SE) | 19.4 (0.5) | 20.0 (0.5) | 19.9 (0.5) | .18 | .30 | .81 |
| SIGMA, estimated marginal means (SE) | 20.2 (0.6) | 20.9 (0.5) | 20.5 (0.5) | .12 | .46 | .31 |
| SSIGP, estimated marginal means (SE) | 19.0 (0.6) | 20.4 (0.5) | 20.0 (0.5) | .009 | .03 | .43 |
| ST, estimated marginal means (SE) | 19.4 (0.6) | 21.4 (0.5) | 20.8 (0.6) | <.001 | .02 | .19 |
| *P*_SIGMA-B vs SIGMA_ | .31 | .18 | .33 | *P*-value  (interaction) | .46 | N/A |
| *P*_SIGMA-B vs SSIGP_ | .53 | .56 | .80 | N/A | N/A | N/A |
| *P*_SIGMA-B vs ST_ | .97 | .050 | .21 | N/A | N/A | N/A |
| *P*_SIGMA vs SSIGP_ | .15 | .45 | .47 | N/A | N/A | N/A |
| *P*_SIGMA vs ST_ | .34 | .53 | .77 | N/A | N/A | N/A |
| *P* _SSIGP vs ST_ | .59 | .16 | .32 | N/A | N/A | N/A |
| Low motivation level |  |  |  |  |  |  |
| SIGMA-Booster, estimated marginal means (SE) | 18.8 (0.6) | 20.0 (0.5) | 20.4 (0.6) | .03 | .01 | .35 |
| SIGMA, estimated marginal means (SE) | 18.3 (0.6) | 19.5 (0.6) | 19.3 (0.5) | .01 | .045 | .60 |
| SSIGP, estimated marginal means (SE) | 18.3 (0.6) | 20.2 (0.6) | 19.5 (0.7) | .001 | .04 | .23 |
| ST, estimated marginal means (SE) | 19.3 (0.6) | 20.0 (0.6) | 18.7 (0.7) | .19 | .36 | .02 |
| *P*_SIGMA-B vs SIGMA_ | .51 | .56 | .15 | *P*-value  (interaction) | .12 | N/A |
| *P*_SIGMA-B vs SSIGP_ | .51 | .77 | .35 | N/A | N/A | N/A |
| *P*_SIGMA-B vs ST_ | .59 | .92 | .07 | N/A | N/A | N/A |
| *P*_SIGMA vs SSIGP_ | .97 | .41 | .80 | N/A | N/A | N/A |
| *P*_SIGMA vs ST_ | .24 | .52 | .49 | N/A | N/A | N/A |
| *P* _SSIGP vs ST_ | .25 | .86 | .42 | N/A | N/A | N/A |
| **Warwick-Edinburgh Mental Well-being Scale** |  |  |  |  |  |  |
| High motivation level |  |  |  |  |  |  |
| SIGMA-Booster, estimated marginal means (SE) | 41.8 (1.0) | 42.6 (1.1) | 44.7 (1.0) | .33 | <.001 | .004 |
| SIGMA, estimated marginal means (SE) | 44.0 (1.1) | 44.2 (1.2) | 45.7 (1.3) | .79 | .16 | .13 |
| SSIGP, estimated marginal means (SE) | 43.1 (1.1) | 44.4 (1.2) | 45.2 (1.2) | .17 | .046 | .53 |
| ST, estimated marginal means (SE) | 42.9 (1.0) | 46.0 (1.2) | 46.7 (1.1) | .002 | <.001 | .46 |
| *P*_SIGMA-B vs SIGMA_ | .13 | .33 | .55 | *P*-value  (interaction) | .41 | N/A |
| *P*_SIGMA-B vs SSIGP_ | .36 | .26 | .74 | N/A | N/A | N/A |
| *P*_SIGMA-B vs ST_ | .44 | .04 | .18 | N/A | N/A | N/A |
| *P*_SIGMA vs SSIGP_ | .59 | .88 | .82 | N/A | N/A | N/A |
| *P*_SIGMA vs ST_ | .47 | .28 | .55 | N/A | N/A | N/A |
| *P* _SSIGP vs ST_ | .88 | .36 | .38 | N/A | N/A | N/A |
| Low motivation level |  |  |  |  |  |  |
| SIGMA-Booster, estimated marginal means (SE) | 42.1 (1.2) | 43.6 (1.4) | 43.3 (1.5) | .13 | .37 | .84 |
| SIGMA, estimated marginal means (SE) | 41.2 (1.2) | 43.0 (1.4) | 43.6 (1.4) | .11 | .06 | .61 |
| SSIGP, estimated marginal means (SE) | 43.2 (1.4) | 43.7 (1.5) | 43.2 (1.4) | .76 | 1.0 | .78 |
| ST, estimated marginal means (SE) | 43.0 (1.2) | 43.6 (1.2) | 45.9 (1.4) | .65 | .04 | .02 |
| *P*_SIGMA-B vs SIGMA_ | .62 | .73 | .89 | *P*-value  (interaction) | .53 | N/A |
| *P*_SIGMA-B vs SSIGP_ | .51 | .97 | .96 | N/A | N/A | N/A |
| *P*_SIGMA-B vs ST_ | .58 | .98 | .20 | N/A | N/A | N/A |
| *P*_SIGMA vs SSIGP_ | .26 | .71 | .85 | N/A | N/A | N/A |
| *P*_SIGMA vs ST_ | .30 | .74 | .25 | N/A | N/A | N/A |
| *P* _SSIGP vs ST_ | .90 | .95 | .17 | N/A | N/A | N/A |

^a^SIGMA-Booster: SIGMA with boosters.

^b^SIGMA: single-session intervention of growth mindset for anxiety.

^c^SSIGP: single-session intervention of growth mindset of personality.

^d^ST: support therapy.
