# Supplementary material for: Effects of Web-Based Single-Session Growth Mindset Interventions for Reducing Adolescent Anxiety: Four-Armed Randomized Controlled Trial
Source: JMIR Pediatr Parent. 2025 Apr 18;8:e63500. doi: 10.2196/63500 (PMC12048788; doi:10.2196/63500)
Supplement: Multimedia Appendix 6 [file pediatrics_v8i1e63500_app6.docx]

**Multimedia Appendix 6**. Moderation effects of baseline growth mindset levels in the treatment effects

| **7-item Generalized Anxiety Disorder** | Baseline | 2-week follow-up | 8-week follow-up | *P*-value  (Baseline  vs 2-week follow-up) | *P*-value  (Baseline vs 8-week follow-up) | *P*-value  (2-week  vs 8-week follow-up) |
| --- | --- | --- | --- | --- | --- | --- |
| Growth mindset |  |  |  |  |  |  |
| SIGMA-Booster^a^, estimated marginal means (SE) | 3.5 (0.3) | 3.5 (0.5) | 3.3 (0.5) | .99 | .66 | .68 |
| SIGMA^b^, estimated marginal means (SE) | 4.3 (0.4) | 4.1 (0.5) | 4.5 (0.5) | .62 | .69 | .32 |
| SSIGP^c^, estimated marginal means (SE) | 4.1 (0.5) | 2.8 (0.4) | 3.2 (0.5) | .002 | .06 | .42 |
| ST^d^, estimated marginal means (SE) | 3.6 (0.4) | 3.0 (0.3) | 3.3 (0.4) | .07 | .47 | .49 |
| *P*_SIGMA-B vs SIGMA_ | .15 | .42 | .10 | *P*-value  (interaction) | .47 | N/A |
| *P*_SIGMA-B vs SSIGP_ | .33 | .29 | .86 | N/A | N/A | N/A |
| *P*_SIGMA-B vs ST_ | .91 | .37 | .97 | N/A | N/A | N/A |
| *P*_SIGMA vs SSIGP_ | .71 | .06 | .07 | N/A | N/A | N/A |
| *P*_SIGMA vs ST_ | .21 | .07 | .06 | N/A | N/A | N/A |
| *P* _SSIGP vs ST_ | .41 | .79 | .88 | N/A | N/A | N/A |
| Fixed mindset |  |  |  |  |  |  |
| SIGMA-Booster, estimated marginal means (SE) | 9.0 (0.5) | 7.5 (0.6) | 7.6 (0.6) | .003 | .01 | .83 |
| SIGMA, estimated marginal means (SE) | 8.9 (0.5) | 7.4 (0.6) | 6.1 (0.5) | .002 | <.001 | .009 |
| SSIGP, estimated marginal means (SE) | 9.1 (0.5) | 7.2 (0.5) | 7.4 (0.5) | <.001 | <.001 | .76 |
| ST, estimated marginal means (SE) | 9.8 (0.5) | 8.1 (0.6) | 8.2 (0.6) | <.001 | .002 | .86 |
| *P*_SIGMA-B vs SIGMA_ | .83 | .88 | .053 | *P*-value  (interaction) | .35 | N/A |
| *P*_SIGMA-B vs SSIGP_ | .94 | .72 | .76 | N/A | N/A | N/A |
| *P*_SIGMA-B vs ST_ | .26 | .50 | .51 | N/A | N/A | N/A |
| *P*_SIGMA vs SSIGP_ | .77 | .84 | .10 | N/A | N/A | N/A |
| *P*_SIGMA vs ST_ | .19 | .42 | .01 | N/A | N/A | N/A |
| *P* _SSIGP vs ST_ | .29 | .30 | .33 | N/A | N/A | N/A |
| **8-item Patient Health Questionnaire** |  |  |  |  |  |  |
| Growth mindset |  |  |  |  |  |  |
| SIGMA-Booster, estimated marginal means (SE) | 4.5 (0.4) | 3.5 (0.5) | 3.2 (0.5) | .11 | .01 | .62 |
| SIGMA, estimated marginal means (SE) | 4.8 (0.4) | 4.0 (0.5) | 4.6 (0.5) | .046 | .61 | .16 |
| SSIGP, estimated marginal means (SE) | 4.9 (0.5) | 2.9 (0.4) | 3.2 (0.5) | <.001 | <.001 | .49 |
| ST, estimated marginal means (SE) | 4.6 (0.4) | 3.2 (0.3) | 3.1 (0.4) | <.001 | .002 | .81 |
| *P*_SIGMA-B vs SIGMA_ | .64 | .47 | .06 | *P*-value  (interaction) | .41 | N/A |
| *P*_SIGMA-B vs SSIGP_ | .58 | .39 | .98 | N/A | N/A | N/A |
| *P*_SIGMA-B vs ST_ | .91 | .67 | .88 | N/A | N/A | N/A |
| *P*_SIGMA vs SSIGP_ | .90 | .10 | .06 | N/A | N/A | N/A |
| *P*_SIGMA vs ST_ | .72 | .19 | .02 | N/A | N/A | N/A |
| *P* _SSIGP vs ST_ | .64 | .58 | .86 | N/A | N/A | N/A |
| Fixed mindset |  |  |  |  |  |  |
| SIGMA-Booster, estimated marginal means (SE) | 9.3 (0.6) | 8.3 (0.6) | 7.6 (0.6) | .06 | .004 | .11 |
| SIGMA, estimated marginal means (SE) | 9.7 (0.5) | 7.8 (0.6) | 6.7 (0.6) | .001 | <.001 | .04 |
| SSIGP, estimated marginal means (SE) | 9.0 (0.6) | 7.7 (0.6) | 7.8 (0.6) | .01 | .01 | .85 |
| ST, estimated marginal means (SE) | 9.9 (0.6) | 8.7 (0.7) | 8.9 (0.6) | .01 | .09 | .76 |
| *P*_SIGMA-B vs SIGMA_ | .62 | .57 | .29 | *P*-value  (interaction) | .23 | N/A |
| *P*_SIGMA-B vs SSIGP_ | .74 | .50 | .81 | N/A | N/A | N/A |
| *P*_SIGMA-B vs ST_ | .41 | .64 | .15 | N/A | N/A | N/A |
| *P*_SIGMA vs SSIGP_ | .39 | .91 | .18 | N/A | N/A | N/A |
| *P*_SIGMA vs ST_ | .73 | .33 | .01 | N/A | N/A | N/A |
| *P* _SSIGP vs ST_ | .24 | .29 | .22 | N/A | N/A | N/A |
| **Suicidal/self-hurting thoughts** |  |  |  |  |  |  |
| Growth mindset |  |  |  |  |  |  |
| SIGMA-Booster, estimated marginal means (SE) | 0.1 (0.04) | 0.2 (0.05) | 0.1 (0.05) | .16 | .46 | .54 |
| SIGMA, estimated marginal means (SE) | 0.2 (0.04) | 0.2 (0.04) | 0.2 (0.04) | .26 | .46 | .65 |
| SSIGP, estimated marginal means (SE) | 0.1 (0.04) | 0.04 (0.03) | 0.01 (0.02) | .15 | .008 | .35 |
| ST, estimated marginal means (SE) | 0.1 (0.04) | 0.2 (0.04) | 0.1 (0.03) | .23 | .29 | .06 |
| *P*_SIGMA-B vs SIGMA_ | .06 | .74 | .60 | *P*-value  (interaction) | .07 | N/A |
| *P*_SIGMA-B vs SSIGP_ | .80 | .02 | .008 | N/A | N/A | N/A |
| *P*_SIGMA-B vs ST_ | .55 | .94 | .44 | N/A | N/A | N/A |
| *P*_SIGMA vs SSIGP_ | .12 | .02 | <.001 | N/A | N/A | N/A |
| *P*_SIGMA vs ST_ | .18 | .65 | .15 | N/A | N/A | N/A |
| *P* _SSIGP vs ST_ | .77 | .005 | .02 | N/A | N/A | N/A |
| Fixed mindset |  |  |  |  |  |  |
| SIGMA-B | 0.5 (0.05) | 0.4 (0.05) | 0.4 (0.05) | .85 | .53 | .70 |
| SIGMA | 0.5 (0.05) | 0.4 (0.05) | 0.4 (0.05) | .10 | .02 | .44 |
| SSIGP | 0.5 (0.06) | 0.4 (0.06) | 0.3 (0.05) | .39 | .008 | .10 |
| ST | 0.4 (0.05) | 0.4 (0.05) | 0.4 (0.05) | .71 | 1.0 | .67 |
| *P*_SIGMA-B vs SIGMA_ | .27 | .95 | .71 | *P*-value  (interaction) | .39 | N/A |
| *P*_SIGMA-B vs SSIGP_ | .74 | .81 | .23 | N/A | N/A | N/A |
| *P*_SIGMA-B vs ST_ | .66 | .54 | .95 | N/A | N/A | N/A |
| *P*_SIGMA vs SSIGP_ | .46 | .85 | .40 | N/A | N/A | N/A |
| *P*_SIGMA vs ST_ | .12 | .58 | .75 | N/A | N/A | N/A |
| *P* _SSIGP vs ST_ | .45 | .73 | .25 | N/A | N/A | N/A |
| **Anxiety Control Questionnaire – Emotion Control** |  |  |  |  |  |  |
| Growth mindset |  |  |  |  |  |  |
| SIGMA-Booster, estimated marginal means (SE) | 16.2 (0.6) | 15.2 (0.6) | 15.2 (0.6) | .16 | .14 | .90 |
| SIGMA, estimated marginal means (SE) | 15.5 (0.5) | 15.2 (0.6) | 15.3 (0.6) | .55 | .74 | .81 |
| SSIGP, estimated marginal means (SE) | 15.8 (0.6) | 16.6 (0.7) | 15.5 (0.7) | .16 | .68 | .13 |
| ST, estimated marginal means (SE) | 16.0 (0.5) | 15.6 (0.6) | 16.0 (0.6) | .53 | .99 | .62 |
| *P*_SIGMA-B vs SIGMA_ | .35 | .93 | .87 | *P*-value  (interaction) | .47 | N/A |
| *P*_SIGMA-B vs SSIGP_ | .69 | .12 | .69 | N/A | N/A | N/A |
| *P*_SIGMA-B vs ST_ | .83 | .66 | .35 | N/A | N/A | N/A |
| *P*_SIGMA vs SSIGP_ | .65 | .10 | .80 | N/A | N/A | N/A |
| *P*_SIGMA vs ST_ | .48 | .58 | .41 | N/A | N/A | N/A |
| *P* _SSIGP vs ST_ | .85 | .25 | .64 | N/A | N/A | N/A |
| Fixed mindset |  |  |  |  |  |  |
| SIGMA-Booster, estimated marginal means (SE) | 12.1 (0.5) | 13.1 (0.4) | 12.5 (0.4) | .04 | .41 | .10 |
| SIGMA, estimated marginal means (SE) | 11.7 (0.4) | 12.6 (0.4) | 11.6 (0.5) | .08 | .94 | .06 |
| SSIGP, estimated marginal means (SE) | 12.3 (0.4) | 12.4 (0.4) | 11.8 (0.5) | .65 | .35 | .18 |
| ST, estimated marginal means (SE) | 10.7 (0.4) | 12.8 (0.5) | 12.8 (0.4) | <.001 | <.001 | .99 |
| *P*_SIGMA-B vs SIGMA_ | .47 | .32 | .14 | *P*-value  (interaction) | .008 | N/A |
| *P*_SIGMA-B vs SSIGP_ | .84 | .20 | .21 | N/A | N/A | N/A |
| *P*_SIGMA-B vs ST_ | .02 | .58 | .62 | N/A | N/A | N/A |
| *P*_SIGMA vs SSIGP_ | .34 | .80 | .82 | N/A | N/A | N/A |
| *P*_SIGMA vs ST_ | .08 | .72 | .07 | N/A | N/A | N/A |
| *P* _SSIGP vs ST_ | .01 | .54 | .11 | N/A | N/A | N/A |
| **Demoralisation Scale - Helplessness** |  |  |  |  |  |  |
| Growth mindset |  |  |  |  |  |  |
| SIGMA-Booster, estimated marginal means (SE) | 7.9 (0.4) | 7.8 (0.4) | 7.6 (0.4) | .84 | .45 | .63 |
| SIGMA, estimated marginal means (SE) | 8.6 (0.4) | 7.5 (0.4) | 8.2 (0.4) | .003 | .37 | .02 |
| SSIGP, estimated marginal means (SE) | 7.0 (0.4) | 7.1 (0.4) | 6.9 (0.4) | .94 | .77 | .76 |
| ST, estimated marginal means (SE) | 7.5 (0.3) | 6.8 (0.3) | 6.7 (0.3) | .06 | .06 | .76 |
| *P*_SIGMA-B vs SIGMA_ | .17 | .58 | .26 | *P*-value  (interaction) | .18 | N/A |
| *P*_SIGMA-B vs SSIGP_ | .07 | .21 | .25 | N/A | N/A | N/A |
| *P*_SIGMA-B vs ST_ | .35 | .07 | .12 | N/A | N/A | N/A |
| *P*_SIGMA vs SSIGP_ | .003 | .43 | .02 | N/A | N/A | N/A |
| *P*_SIGMA vs ST_ | .02 | .16 | .004 | N/A | N/A | N/A |
| *P* _SSIGP vs ST_ | .33 | .66 | .73 | N/A | N/A | N/A |
| Fixed mindset |  |  |  |  |  |  |
| SIGMA-Booster, estimated marginal means (SE) | 11.0 (0.3) | 10.8 (0.4) | 10.6 (0.4) | .50 | .15 | .33 |
| SIGMA, estimated marginal means (SE) | 11.8 (0.3) | 10.6 (0.4) | 10.0 (0.4) | .02 | <.001 | .24 |
| SSIGP, estimated marginal means (SE) | 11.3 (0.4) | 10.6 (0.4) | 10.3 (0.3) | .07 | .003 | .50 |
| ST, estimated marginal means (SE) | 12.0 (0.4) | 10.8 (0.4) | 11.4 (0.4) | .001 | .06 | .20 |
| *P*_SIGMA-B vs SIGMA_ | .12 | .73 | .33 | *P*-value  (interaction) | .16 | N/A |
| *P*_SIGMA-B vs SSIGP_ | .56 | .59 | .60 | N/A | N/A | N/A |
| *P*_SIGMA-B vs ST_ | .04 | .98 | .16 | N/A | N/A | N/A |
| *P*_SIGMA vs SSIGP_ | .36 | .88 | .61 | N/A | N/A | N/A |
| *P*_SIGMA vs ST_ | .60 | .73 | .03 | N/A | N/A | N/A |
| *P* _SSIGP vs ST_ | .16 | .61 | .06 | N/A | N/A | N/A |
| **Attitude towards Seeking Help** |  |  |  |  |  |  |
| Growth mindset |  |  |  |  |  |  |
| SIGMA-Booster, estimated marginal means (SE) | 20.0 (0.6) | 20.6 (0.6) | 20.6 (0.6) | .27 | .30 | .85 |
| SIGMA, estimated marginal means (SE) | 20.5 (0.6) | 21.3 (0.6) | 20.5 (0.6) | .08 | .90 | .04 |
| SSIGP, estimated marginal means (SE) | 19.4 (0.7) | 20.4 (0.7) | 20.7 (0.8) | .08 | .045 | .69 |
| ST, estimated marginal means (SE) | 20.8 (0.6) | 21.8 (0.6) | 20.6 (0.7) | .09 | .67 | .03 |
| *P*_SIGMA-B vs SIGMA_ | .58 | .34 | .88 | *P*-value  (interaction) | .38 | N/A |
| *P*_SIGMA-B vs SSIGP_ | .53 | .90 | .97 | N/A | N/A | N/A |
| *P*_SIGMA-B vs ST_ | .36 | .15 | .92 | N/A | N/A | N/A |
| *P*_SIGMA vs SSIGP_ | .25 | .35 | .87 | N/A | N/A | N/A |
| *P*_SIGMA vs ST_ | .68 | .56 | .97 | N/A | N/A | N/A |
| *P* _SSIGP vs ST_ | .14 | .18 | .91 | N/A | N/A | N/A |
| Fixed mindset |  |  |  |  |  |  |
| SIGMA-Booster, estimated marginal means (SE) | 18.7 (0.4) | 19.7 (0.5) | 19.8 (0.4) | .02 | .01 | .77 |
| SIGMA, estimated marginal means (SE) | 18.1 (0.5) | 19.2 (0.5) | 19.4 (0.4) | .03 | .01 | .61 |
| SSIGP, estimated marginal means (SE) | 18.2 (0.5) | 20.2 (0.4) | 19.3 (0.4) | <.001 | .03 | .02 |
| ST, estimated marginal means (SE) | 18.0 (0.5) | 19.9 (0.4) | 19.2 (0.5) | .001 | .047 | .15 |
| *P*_SIGMA-B vs SIGMA_ | .34 | .44 | .49 | *P*-value  (interaction) | .32 | N/A |
| *P*_SIGMA-B vs SSIGP_ | .43 | .37 | .37 | N/A | N/A | N/A |
| *P*_SIGMA-B vs ST_ | .26 | .75 | .36 | N/A | N/A | N/A |
| *P*_SIGMA vs SSIGP_ | .89 | .08 | .84 | N/A | N/A | N/A |
| *P*_SIGMA vs ST_ | .93 | .27 | .76 | N/A | N/A | N/A |
| *P* _SSIGP vs ST_ | .82 | .57 | .90 | N/A | N/A | N/A |
| **Warwick-Edinburgh Mental Well-being Scale** |  |  |  |  |  |  |
| Growth mindset |  |  |  |  |  |  |
| SIGMA-Booster, estimated marginal means (SE) | 46.1 (1.3) | 46.6 (1.5) | 46.6 (1.5) | .69 | .75 | .98 |
| SIGMA, estimated marginal means (SE) | 46.4 (1.2) | 46.8 (1.3) | 48.4 (1.4) | .68 | .15 | .20 |
| SSIGP, estimated marginal means (SE) | 48.4 (1.4) | 48.3 (1.7) | 49.2 (1.7) | .96 | .65 | .68 |
| ST, estimated marginal means (SE) | 48.3 (1.1) | 48.9 (1.2) | 51.4 (1.2) | .55 | .002 | .01 |
| *P*_SIGMA-B vs SIGMA_ | .86 | .88 | .38 | *P*-value  (interaction) | .75 | N/A |
| *P*_SIGMA-B vs SSIGP_ | .22 | .41 | .26 | N/A | N/A | N/A |
| *P*_SIGMA-B vs ST_ | .20 | .22 | .01 | N/A | N/A | N/A |
| *P*_SIGMA vs SSIGP_ | .27 | .48 | .72 | N/A | N/A | N/A |
| *P*_SIGMA vs ST_ | .24 | .26 | .11 | N/A | N/A | N/A |
| *P* _SSIGP vs ST_ | .94 | .77 | .31 | N/A | N/A | N/A |
| Fixed mindset |  |  |  |  |  |  |
| SIGMA-Booster, estimated marginal means (SE) | 39.1 (0.8) | 40.7 (0.9) | 42.4 (0.9) | .051 | <.001 | .046 |
| SIGMA, estimated marginal means (SE) | 38.7 (0.9) | 40.1 (1.1) | 40.7 (1.1) | .11 | .06 | .53 |
| SSIGP, estimated marginal means (SE) | 39.5 (0.9) | 41.2 (1.0) | 41.1 (0.8) | .09 | .07 | .93 |
| ST, estimated marginal means (SE) | 38.3 (0.9) | 41.4 (1.1) | 41.9 (1.1) | .006 | .002 | .60 |
| *P*_SIGMA-B vs SIGMA_ | .74 | .67 | .22 | *P*-value  (interaction) | .60 | N/A |
| *P*_SIGMA-B vs SSIGP_ | .75 | .71 | .26 | N/A | N/A | N/A |
| *P*_SIGMA-B vs ST_ | .51 | .61 | .72 | N/A | N/A | N/A |
| *P*_SIGMA vs SSIGP_ | .54 | .46 | .76 | N/A | N/A | N/A |
| *P*_SIGMA vs ST_ | .75 | .40 | .43 | N/A | N/A | N/A |
| *P* _SSIGP vs ST_ | .36 | .89 | .55 | N/A | N/A | N/A |

^a^SIGMA-Booster: SIGMA with boosters.

^b^SIGMA: single-session intervention of growth mindset for anxiety.

^c^SSIGP: single-session intervention of growth mindset of personality.

^d^ST: support therapy.
